# Reversible solvatomagnetic switching in a single-ion magnet from an entatic state†
†Electronic supplementary information (ESI) available: Preparation methods and physical characterization data. Crystallographic refinement and computational details. Additional figures (Fig. S1–S12) and tables (Tables S1–S5). CCDC 952077 and 938463. For ESI and crystallographic data in CIF or other electronic format see DOI: 10.1039/c6sc05188j



**DOI:** 10.1039/c6sc05188j

**Published:** 2017-02-13

**Authors:** J. Vallejo, E. Pardo, M. Viciano-Chumillas, I. Castro, P. Amorós, M. Déniz, C. Ruiz-Pérez, C. Yuste-Vivas, J. Krzystek, M. Julve, F. Lloret, J. Cano

**Affiliations:** a Institut de Ciència Molecular (ICMOL) , Universitat de València , 46980 Paterna , València , Spain . Email: joan.cano@uv.es ; Email: Emilio.Pardo@uv.es; b Institut de Ciència del Materials (ICMUV) , Universitat de València , 46980 Paterna , València , Spain; c Laboratorio de Rayos X y Materiales Moleculares , Departamento de Física , Facultad de Ciencias (Sección Física) , Universidad de La Laguna , 38201 Tenerife , Spain; d National High Magnetic Field Laboratory (NHMFL) , Florida State University , Tallahassee , Florida 32310 , USA; e Fundació General de la Universitat de València (FGUV) , Spain

## Abstract

A vast impact on molecular nanoscience can be achieved using simple transition metal complexes as dynamic chemical systems to perform specific and selective tasks under the control of an external stimulus that switches “ON” and “OFF” their electronic properties. While the interest in single-ion magnets (SIMs) lies in their potential applications in information storage and quantum computing, the switching of their slow magnetic relaxation associated with host–guest processes is insufficiently explored. Herein, we report a unique example of a mononuclear cobalt(ii) complex in which geometrical constraints are the cause of easy and reversible water coordination and its release. As a result, a reversible and selective colour and SIM behaviour switch occurs between a “slow-relaxing” deep red anhydrous material (compound **1**) and its “fast-relaxing” orange hydrated form (compound **2**). The combination of this optical and magnetic switching in this new class of vapochromic and thermochromic SIMs offers fascinating possibilities for designing multifunctional molecular materials.

## Abstract 2

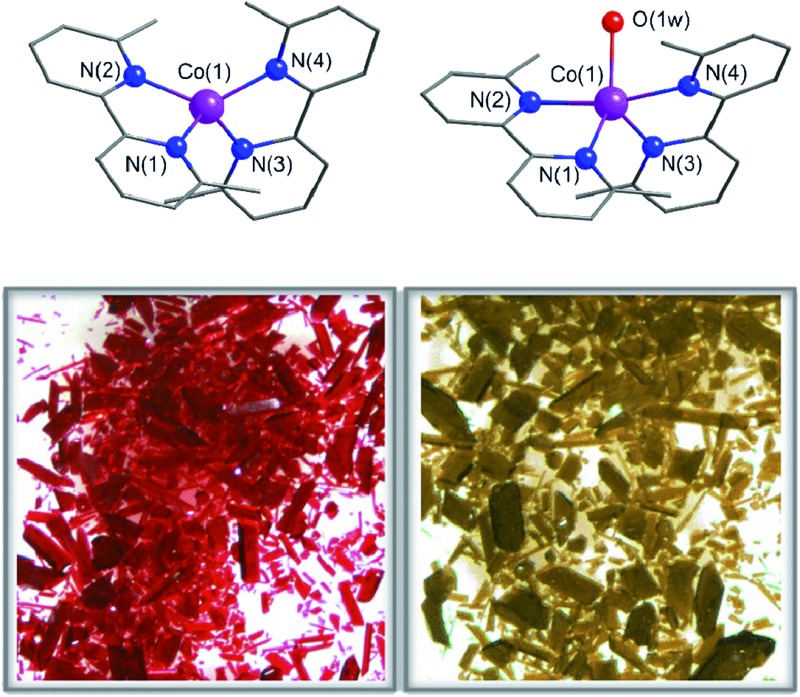
We have developed a new strategy for the design and synthesis of multifunctional molecular materials showing reversible magnetic and optical switching.

## Introduction

The first observation in 1993 of slow magnetic relaxation and hysteretic effects in high-nuclearity metal complexes constituted a milestone in the field of molecular nanomagnetism.^1^ The idea that a single molecule may show magnetic hysteresis was formulated by O. Kahn only two years before,^2^ initiating a debate concerning the possibility of hysteresis at the purely molecular scale in an assembly of magnetically isolated molecules. The following decade after this discovery saw increasing attention paid to the polynuclear complexes of a variety of transition metal ions as examples of so-called single-molecule magnets (SMMs).^3^ Besides the interest in SMMs as test models of quantum mechanical effects,^4^ they are also relevant because of their potential applications in high-density magnetic memories or quantum computing devices.^5–8^ The discovery in 2003 of slow magnetic relaxation effects in highly anisotropic mononuclear rare-earth complexes renewed the debate in the SMM area.^9,10^ Long after this pioneering work, the mere existence of this property in related mononuclear transition metal complexes was still questioned. However, subsequent studies on some high-spin iron(ii),^11–14^ cobalt(ii),^15–23^ rhenium(iv),^24,25^ manganese(iii),^26–28^ iron(i),^29^ nickel(ii)^30^ and low-spin nickel(i)^31^ mononuclear complexes exhibiting slow magnetic relaxation provided a clear-cut answer, thus closing the debate about the possibility of mononuclear complexes showing this property.

Molecules with a single slow-relaxing metal centre, referred to as single-ion magnets (SIMs), are the simplest model systems for fundamental research on magnetic anisotropy and magnetic relaxation dynamics, from both experimental and theoretical points of view.^32,33^ Being a non-cooperative property of an isolated molecule, the SIM behaviour depends on subtle and small structural variations in the metal coordination environment that directly determine the nature and magnitude of the local single-ion magnetic anisotropy, *e.g.* peripheral ligand modifications.^16^ SIMs are also particularly appealing because the slow magnetic relaxation effects can be controlled with structural modulation and an external stimulus (*e.g.*, a magnetic field). These features together with their small size and easy handling and addressing to surfaces make these simple switchable molecules candidates for new magnetic devices in molecular electronics and spintronics.

The “entatic state” is a common term in biology, but is only rarely used in other disciplines such as bioinorganic or coordination chemistry.^34–36^ It is typically found in the activity of some enzymes, specifically those controlling redox processes. An “entatic state” is related to a constrained geometry halfway between the ideal geometries at the beginning and end of a reaction. The energetic requirements for a chemical reaction, *i.e.* the energy barriers, are minimized in these cases and the reaction is favoured. In other cases, the uptake and release of the substrate molecules are controlled by the entatic states. An illustrative example is provided by iron and manganese superoxide dismutases (SODs), where a distortion of the trigonal bipyramidal geometry (TBPY-5) of the active site provides easy entry for the charged superoxide (O_2_^–^) substrate. After evolution to hydrogen peroxide or molecular oxygen, the active site adopts a strongly distorted octahedral (OC-6) geometry that largely limits the space available to accommodate the generated neutral substrates (H_2_O_2_ and O_2_), which are minimized in these cases easily released because the Coulomb attraction between these substrates and the metal ion vanishes. Recently, the entatic state concept has also been indirectly used for “local sterically constrained environments” to improve new hybrid solar thermal fuels through the preservation of the robust cyclability and stability of the material.^37^

With the design and synthesis of multifunctional molecular materials for magnetic (or optical) switching in mind,^38–41^ we have developed a new strategy based on mononuclear cobalt(ii) complexes with β,β′-dimethyl-substituted aromatic α,α′-diimines, such as 6,6′-dimethyl-2,2′-bipyridine (dmbpy) and 2,9-dimethyl-1,10-phenanthroline (dmphen). This class of sterically hindered, chelating ligands causes important structural distortions of the metal environment that can ultimately lead to a large single-ion magnetic anisotropy.^17^ Along this line, we report herein the synthesis, X-ray structures, thermal and sorption behaviours, spectroscopic and magnetic properties, and theoretical calculations of two new mononuclear cobalt(ii) complexes with formulas [Co^II^(dmbpy)_2_](ClO_4_)_2_ (**1**) and [Co^II^(dmbpy)_2_(H_2_O)](ClO_4_)_2_ (**2**). This pair of anhydrous (**1**) and hydrated (**2**) forms exhibits a unique vapour- and thermochromic-induced SIM switching in the solid state, which is accompanied by reversible changes in their colour and magnetic properties. This switching behaviour is related to the coordination and release of a water molecule through vapour adsorption and heating, reminiscent of classical cobalt(ii)-substituted molecular sieves (*e.g.* aluminophosphates or silicates like the well-known silica gel).^42^ The reversibility of this process is connected to a large distortion in both complexes, which is associated with entatic states induced by the steric constrains caused by the bulky chelating ligands.

## Results and discussion

### X-ray crystal structure

**Fig. 1:**
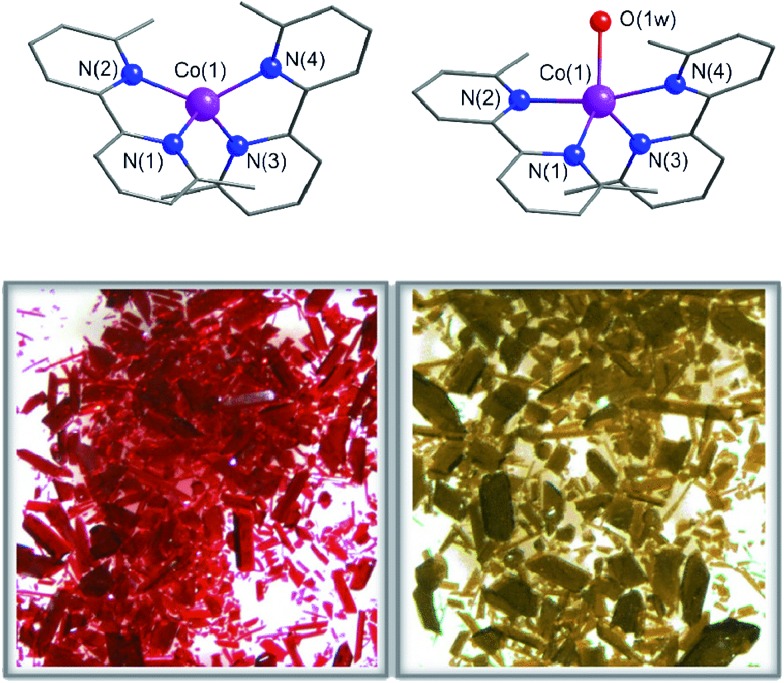
Top: Perspective views of the mononuclear cobalt(ii) units of **1** (left) and **2** (right). Purple, blue and red spheres represent Co, N and O atoms, respectively. The organic skeleton built with C atoms is shown in grey and H atoms are omitted for clarity. Bottom: Optical microscopy images of a bunch of single crystals that show the colour changes that occur upon leaving **1** in the open air (right) or after heating **2** at 75 °C (left).

Compounds **1** and **2** crystallize in the monoclinic *P*2_1_/*c* space group with minor variations in the unit cell parameters that lead to a slightly larger unit cell volume in **2**. The crystal structures of **1** and **2** consist of either bis(6,6′-dimethyl-2,2′-bipyridine)cobalt(ii) or bis(6,6′-dimethyl-2,2′-bipyridine)(aqua)cobalt(ii) cations, [Co^II^(dmbpy)_2_]^2+^ (**1**) and [Co^II^(dmbpy)_2_(H_2_O)]^2+^ (**2**), with a pseudo two-fold molecular symmetry and non-coordinated perchlorate counteranions (Fig. 1). The mononuclear cobalt units are rather well separated from each other in the crystal lattice with the shortest intermetallic distances of 7.809(1) (**1**) and 8.6055(10) Å (**2**) (Fig. S1, ESI†). Although there are some weak intermolecular π–π stacking interactions between the pyridyl rings of the chelating dmbpy ligands of neighbouring units in **1** and **2** [inter-plane distances equal to 3.63 Å (**1**) and 3.83 Å (**2**)] (Fig. S1a, ESI†), only supramolecular pairs occur. Weak hydrogen bonding interactions between the coordinated water molecule and the neighbouring perchlorate anions are also found in **2** [O···Ow = 2.723(7)–2.929(9) Å and O···H = 1.84(6)–2.08(6) Å], but they do not connect different Co^II^ complexes (Fig. S1b, ESI†).

Each cobalt atom in **1** is four-coordinate in a somewhat distorted tetrahedral (T-4) CoN_4_ environment, whereas it is five-coordinate in **2** adopting a CoN_4_Ow coordination sphere that is intermediate between a square pyramid (SPY-5) and a trigonal bipyramid (TBPY-5) (Fig. 1). The so-called Berry pseudo-rotation parameter, defined as *τ* = |*α* – *β*|/60, where *α* and *β* are the largest LCoL angles, is 0.44 for **2** (*τ* is equal to 1 and 0 for ideal TBP and SQP geometries).^43^ Nevertheless, this parameter is not the most adequate way to describe the highly distorted environment in **2**, which is closer to a distorted asymmetric T path with a N(2)Co(1)N(4) angle (*θ*) equal to 165.8° and two different *γ* angles, 115.2 and 139.4° for OwCo(1)N(4) and OwCo(1)N(1), respectively. The tetrahedron in **1** is defined by four imine-nitrogen atoms from two almost perpendicularly placed chelating dmbpy ligands (Fig. 1), with the angle (*α*) between the Co(1)N(1)N(2) and Co(1)N(3)N(4) planes equal to 88.7(2)° and close to those found in a few very similar complexes (86.5° and 81.5°).^44,45^ As in these other cases, the bite angles from the two chelating dmbpy ligands [N(1)–Co(1)–N(2) = 83.5(2)° and N(3)–Co(1)–N(4) = 83.02(2)°] and the remaining free interbond angles [N–Co–N = 115.7(2)–131.4(2)°] in **1** are smaller and larger, respectively, than those for an ideal tetrahedron (109.5°). In contrast, it is very difficult to assign the axial and equatorial positions in **2** due to the highly distorted metal environment intermediate between a SQP and TBP (Fig. 1). Overall, a “plier” distortion in **1** leads to pseudo *D*_2d_ symmetry,^46^ whereas the symmetry of **2** is halfway between *C*_4v_ and *D*_3h_. This rare geometry for **2** is a consequence of the high geometrical restrictions that avoid a relaxing geometry when the water molecule is removed. As a result, continuous shape measurements^47^ (CShM) relative to the tetrahedron (T-4) and square plane (SP-4) on CoN_4_ coordination spheres are similar for **1** (5.2 and 25.7°) and for the dehydrated form of **2** (**2deh**, 9.4 and 18.3°). Moreover, quasi-coincident deviations from the pathway connecting the two ideal polyhedra were found for **1** and **2deh** (23.9 and 22.4°, respectively).

### Chemical interconversion and physical characterization

**Fig. 2:**
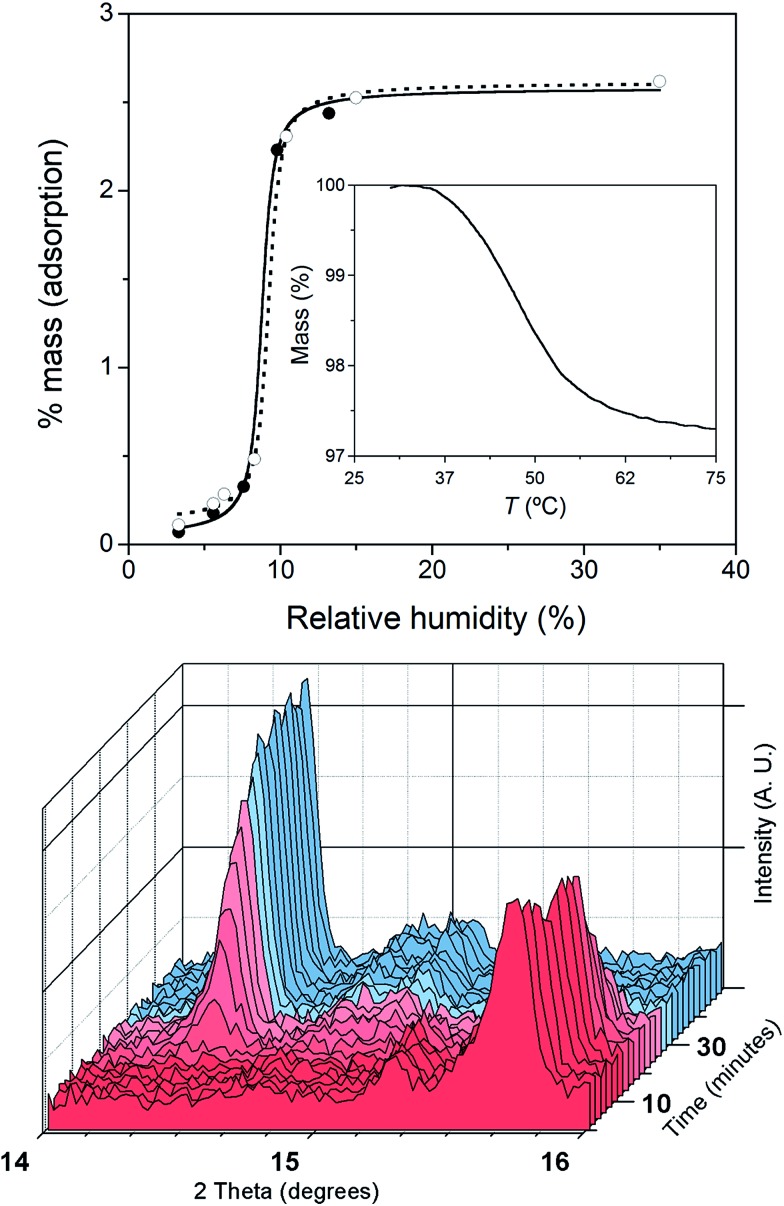
Top: The water vapour adsorption () and desorption (○) isotherms of **1** at 25 °C; the solid and dashed lines correspond to the smoothed curves for the adsorption and desorption regimes. The inset shows the release of the coordinated water molecule of **2** under dry N_2_. Bottom: XRPD patterns showing the structural changes that occur in a crystalline powdered sample of **1** after leaving it in open air to give the hydrated compound **2**.

The crystals of **1** were unstable when separated from the mother liquor and had to be stored under Ar to prevent the transformation into **2** through slow water uptake in the open air. Conversely, the crystals of **2** are air stable at room temperature but they lose the coordinated water under gentle warming to afford the anhydrous derivative **1**. These transformations were accompanied by substantial colour changes from dark red (**1**) to bright orange (**2**) and *vice versa* (Fig. 1). In fact, the thermogravimetric analysis (TGA) of a crystalline powdered sample of **2** in a dry N_2_ atmosphere showed a rapid mass loss (*ca.* 2.8%) from room temperature at 75 °C, which corresponds exactly to the release of the coordinated water molecule (inset in Fig. 2). No other changes occur at higher temperatures until 300 °C, when the decomposition of the anhydrous phase starts (Fig. S2a†). Conversely, an abrupt and total recovery of the initial mass of **2** was observed under a weak relative humidity (RH *ca.* 10%), suggesting the occurrence of moderate host–guest interactions, as expected for the direct coordination of guest water to the host metal centres. Lastly, the water vapour absorption/desorption isotherms of a crystalline powdered sample of **1** at 25 °C also confirmed that the rehydration/dehydration process is reversible, complete, and with no hysteresis (Fig. 2).

**Fig. 3:**
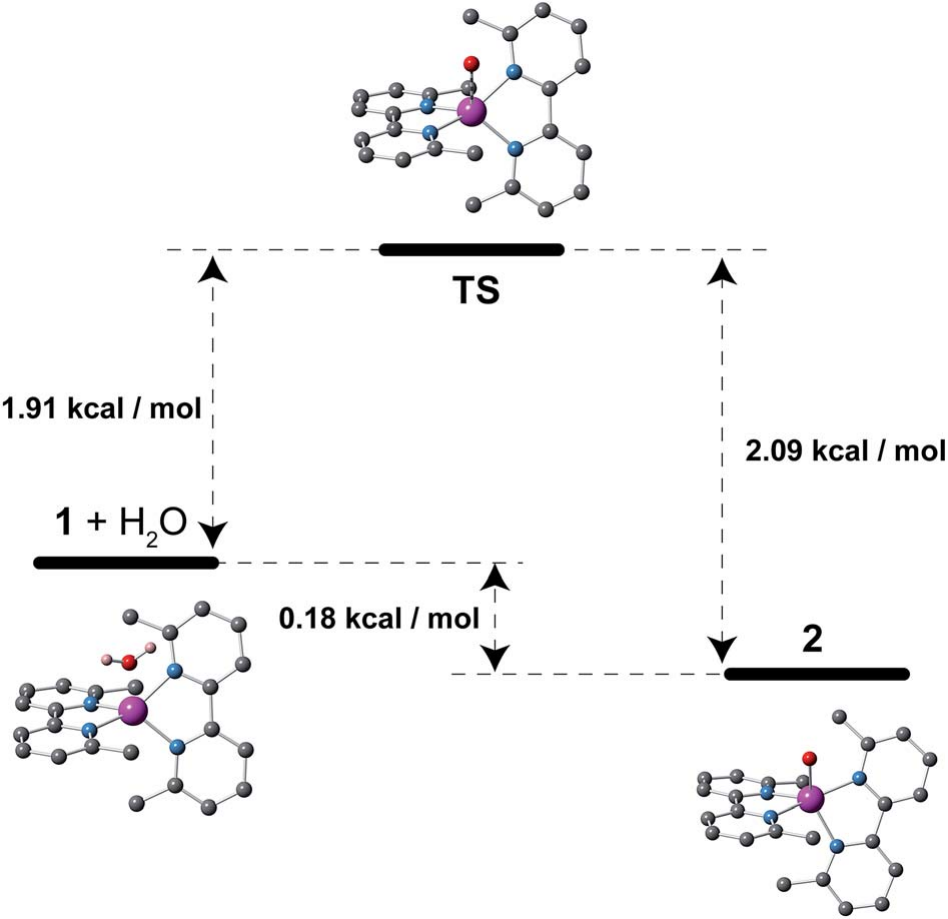
Schematic view of the comparative stability of the species involved in the dehydration process: hydrated and dehydrated forms and the transition state (TS) for the water release. The calculated relative free energies (in kcal mol^–1^) at 298 K in the gas phase and views of the optimized molecular geometries are also shown. Purple, red, blue, grey and pink spheres correspond to the cobalt, oxygen, nitrogen, carbon and hydrogen atoms, respectively. Most of the hydrogen atoms are not shown for clarity.

This reversibility is linked to the highly distorted geometries of the metal environments in **1** and **2**, which clearly favour the easy and reversible entry and release of a water molecule into the cobalt coordination sphere. As with the iron and manganese SODs, there are two entatic states that minimize the energy barrier for the entry and release of the water molecule, converting this into a reversible process. The structural modifications of the Co^II^ complex accompanying the dehydration/rehydration process are quite small (Fig. 1 and S1, ESI†) – as was already established from the continuous shape measurements – which accounts for the modest value of the energy barriers for each step in the dehydration/rehydration process and explains its reversibility. Accordingly, in agreement with the experimental observation and from the free energies calculated in the gas phase, the hydrated form is the most stable with an energy barrier of just 0.18 kcal mol^–1^ for the hydration process (Fig. 3). Even if the energy barrier for the dehydration is larger (2.09 kcal mol^–1^), it is small enough to remove the coordinated water molecule with mild heating.

The successive heating and exposure to air of both a polycrystalline sample and a solution of **2** were followed up with various spectroscopic techniques corroborating the reversible uptake and release of a water molecule from the metal coordination sphere. The XRPD patterns of the dehydrated and rehydrated solid samples, in perfect agreement with the theoretical patterns of **1** and **2** (Fig. S2, ESI†), showed significant shifts of the peaks. The time-dependent XRPD patterns showed that, after a short induction period of approximately 10 min, half and complete conversions were reached in 20 and 60 min, respectively (Fig. 2), retaining the crystallinity of the sample in both phases after five dehydration/rehydration cycles.

**Fig. 4:**
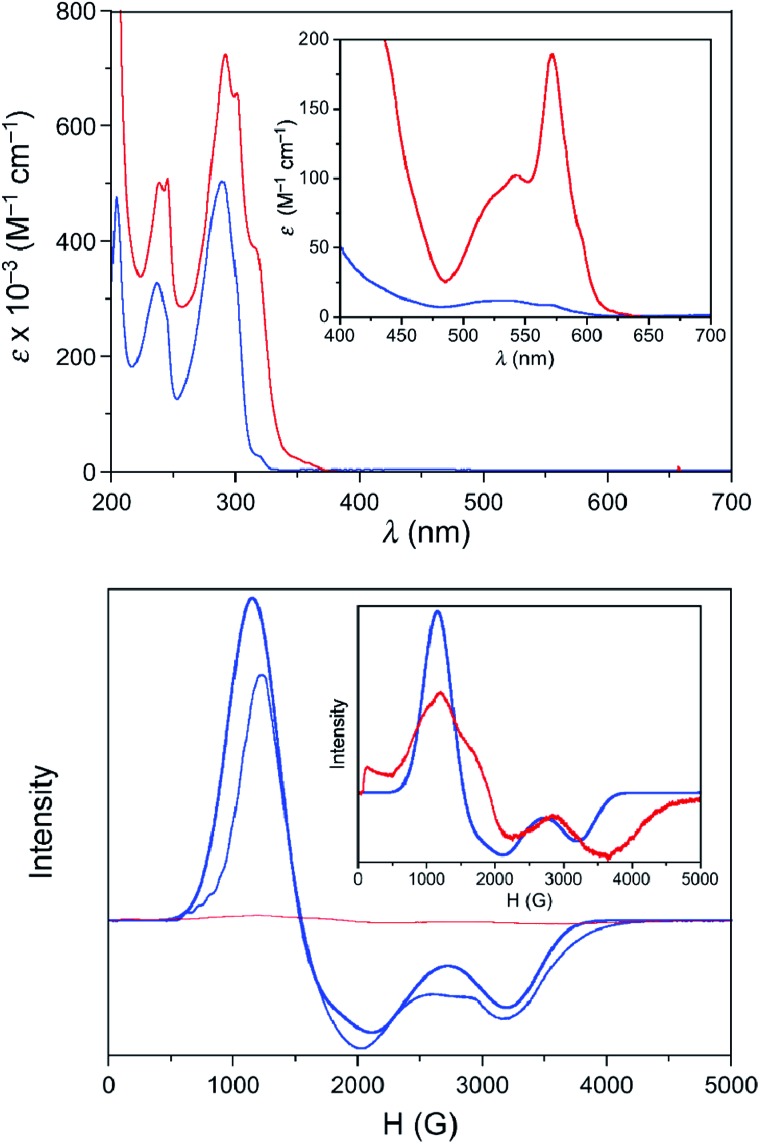
Top: UV-Vis spectra at room temperature of solution samples of **1** (acetonitrile solution, red solid line) and **2** (methanol solution, blue solid line). Magnification in the visible region is shown in the inset. Bottom: X-band EPR spectra of solution samples of 1 (red) and 2 (blue) at 4.0 K. The bold line corresponds to the best simulation of the EPR data of **2** with *g*_1_ = 2.10, *g*_2_ = 4.05, and *g*_3_ = 5.54. Magnification of the experimental EPR spectrum of **1** together with the simulated spectrum of **2** are displayed in the inset.

**Fig. 5:**
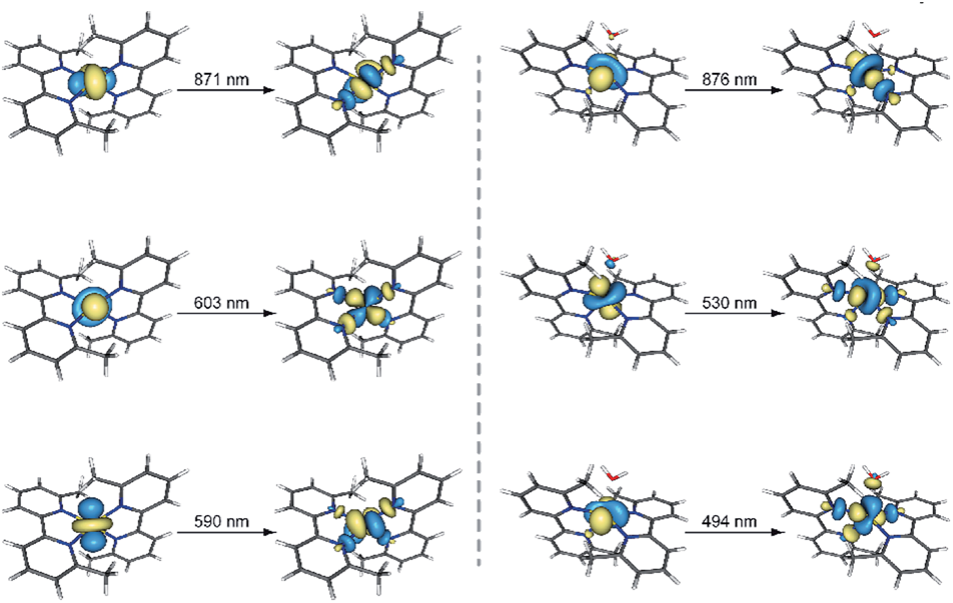
Perspective views of the natural transition orbitals (NTOs) involved in the theoretical electronic excitations shown in Table S3† for **1** (left) and **2** (right). NTOs have been used because they provide an easier visualization of the electronic transitions compared to molecular orbitals. The isodensity surfaces correspond to a cut-off value of 0.03 e bohr^–3^. In this picture, one electron is promoted from the orbital at the left side to the other one at the right side.

The UV-Vis spectra in the solid state and in solution of the dehydrated and rehydrated samples matched with those of **1** and **2**, supporting the assumption that the changes upon the reversible dehydration/rehydration process occur in the metal coordination sphere (Fig. 4 and S3a, ESI†). Both compounds showed the same electronic absorption spectra for the high energy region (200–300 nm), with two very intense and sharp intraligand bands. Each of the two compounds also exhibits a weaker and asymmetric band in the visible region although the intensity and energy of this band differs moderately, reflecting the changes in the metal environment. The deconvolution of the visible-region band in the solution spectra showed that it is composed of a set of electronic transitions (Table S3, ESI†). The fact that some of the electronic transitions in both compounds have the same energy suggests the presence of a mixture of the hydrated and dehydrated species. Nevertheless, the most intense electronic transitions have lower energies and are largely more intense in **1** (548, 572 and 588 nm) than in **2** (527 nm). These features agree with d–d transitions in the T-4 and SPY-5 ligand fields for **1** and **2**, respectively. In this respect, it is well known that more intense and less energetic electronic transitions are provided by a less symmetric and weaker T-4 ligand field. Despite a slight shift in energy from the experimental data, TDDFT calculations in acetonitrile solution on both complexes supported the experimental observation, reproducing the relative positions and intensities of the electronic transitions in the discussed energy region. Besides, the TDDFT calculations predicted only two d–d electronic transitions for the hydrated and dehydrated species in the 500–600 nm region (Table S3, ESI†), as can be easily seen from the involved natural transition orbitals (Fig. 5). This is a confirmation that the experimental spectra in solution correspond to a mixture of the hydrated and dehydrated species with one of them being predominant, rather than to a single compound.

### HFEPR spectroscopy and static (dc) magnetic properties

The presence of a zero-field splitting (zfs) is typical in high-spin cobalt(ii) complexes, the magnitude of which is usually very large (on the order of 100 cm^–1^) in OC-6 complexes because of a first-order spin–orbit coupling (SOC). In ideal or quasi-ideal T-4 geometries, a second-order SOC contribution is responsible for a more moderate but non-negligible zfs (typically on the order of 10 cm^–1^). In both cases, the quartet (*S* = 3/2) ground spin state is split into two multiplets corresponding to *M*_S_ = ±3/2 and ±1/2 (so-called Kramers doublets) in the absence of a magnetic field. The generally very large zfs in OC-6 complexes precludes its determination using the most accurate technique, high-frequency and -field electron paramagnetic resonance (HFEPR), as the available frequencies and magnetic field are not sufficient to couple the spin sublevels belonging to the two different Kramers doublets. Instead, the allowed Δ*M*_S_ = ±1 transition between the |–1/2〉 and |+1/2〉 levels of the *M*_S_ = |±1/2〉 manifold is routinely observed if that manifold lies lower on the energy scale than the *M*_S_ = |±3/2〉 one, *i.e.* when *D* > 0. Although this transition is not informative with regard to the magnitude of *D*, it allows one to confirm its sign and also evaluate the zfs rhombicity factor *E*/*D*. In the reverse case of *D* < 0, the lower-lying Kramers doublet is *M*_S_ = |±3/2〉, and the intra-Kramers EPR transition within this multiplet corresponds to Δ*M*_S_ = ±3 which is nominally forbidden. Such systems (large negative *D*, and *E* = 0) are “EPR-silent”.^48^ The transition in question becomes partly allowed in the presence of the zfs *E*-term, which mixes the |–3/2〉 and |+3/2〉 levels. Its presence (or absence) is thus a sensitive probe of the rhombicity of the zfs tensor.

**Fig. 6:**
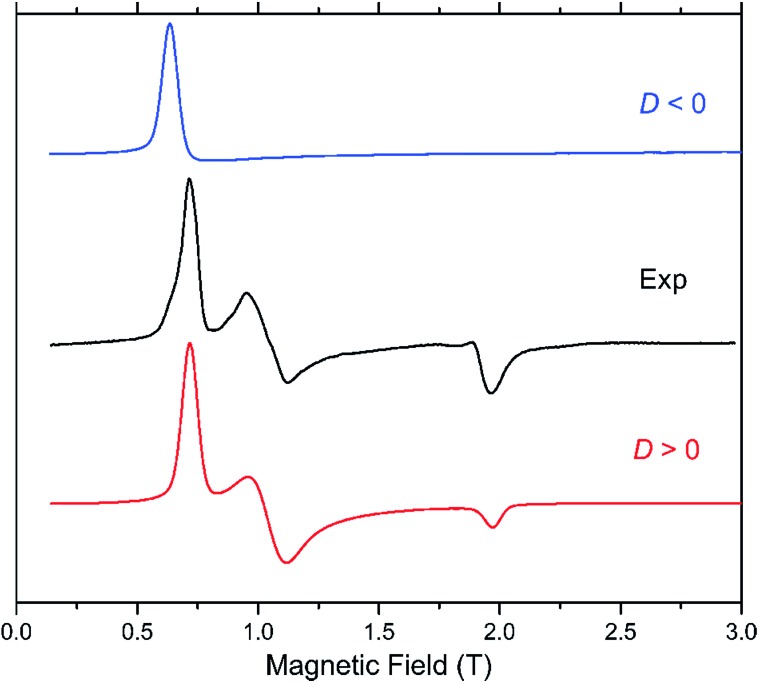
HFEPR spectrum of **2** recorded at 4.5 K and 56 GHz (black trace). Simulations using *S* = 3/2, |*D*| = 28 cm^–1^ (corresponding to the result of NEVPT2 calculations), |*E*/*D*| = 0.123, *g*_⊥_ = 2.37 and *g*_∥_ = 2.12 are in blue and red traces for a positive and negative axial *D* parameter, respectively. **1** is EPR-silent in the frequency range from 52 to 610 GHz.

In our case, complex **1** belongs to the HFEPR-silent category, in qualitative agreement with the large negative *D* (–57.0 cm^–1^) and very low *E*/*D* (0.004) values estimated from NEVPT2 calculations. Unlike in **1**, EPR spectra were observed for **2** in the 52–320 GHz frequency range which correspond to those expected for an *S* = 3/2 spin state with a positive *D* and sizeable rhombic ratio *E*/*D*. The pattern does not depend on frequency, thus the condition *hν* ≪ |*D*| (*ν* is the Larmor frequency) is satisfied for the whole frequency range. Fig. 6 shows one such spectrum at 56 GHz and low temperature (4.5 K) together with simulations that assume the magnitude of |*D*| obtained from NEVPT2 calculations. It is clear that the sign of *D* is positive. The spectrum at a much higher frequency (319 GHz) and its simulations are shown in Fig. S4 in the ESI.† An analysis of the multifrequency set of resonances (Fig. S5, ESI†) yields a moderate rhombicity (*E*/*D* = 0.125) of the zfs tensor together with an axial Landé *g*-tensor: *g*_⊥_ = 2.37 and *g*_∥_ = 2.16. These results agree with those obtained from the NEVPT2 calculations (*E*/*D* = 0.198), with the rhombicity *E*/*D* factor being slightly overestimated by the theory, as usually occurs in most cobalt(ii) cases.

Because of the limited access to the HFEPR technique, successive dehydration/rehydration processes on a sample of **2** were followed using X-band EPR spectroscopy at 4.0 K. Whereas **2** shows a rhombic signal that could be analysed as a doublet effective spin (*S*_eff_ = 1/2), with *g*_1_ = 2.10, *g*_2_ = 4.05, and *g*_3_ = 5.54 in solution (Fig. 4) and in the solid state (Fig. S3b, ESI†), no signal was recorded for **1** yet again. To be exact, despite all the care taken, and in accordance with the easy hydration of **1** observed from the XPRD studies and UV-Vis electronic spectroscopy, a zoom of the EPR spectra of **1** in solution confirmed partial hydration to afford **2** but in a very low amount (less than 2 per cent). These *g*_*i*_ components for an effective spin agree well with those deduced from the *g*-tensor and *E*/*D* values found in the HFEPR study for *S* = 3/2 using *D* = +28 cm^–1^ obtained from NEVPT2 calculations (*g*_*x*_ = 5.58, *g*_*y*_ = 3.88, and *g*_*z*_ = 2.07).^49^ Like for other spectroscopic techniques, the EPR spectra successively matched up with those of **1** and **2** after several dehydration/rehydration cycles, confirming the reversibility of the uptake/release of a water molecule.

**Fig. 7:**
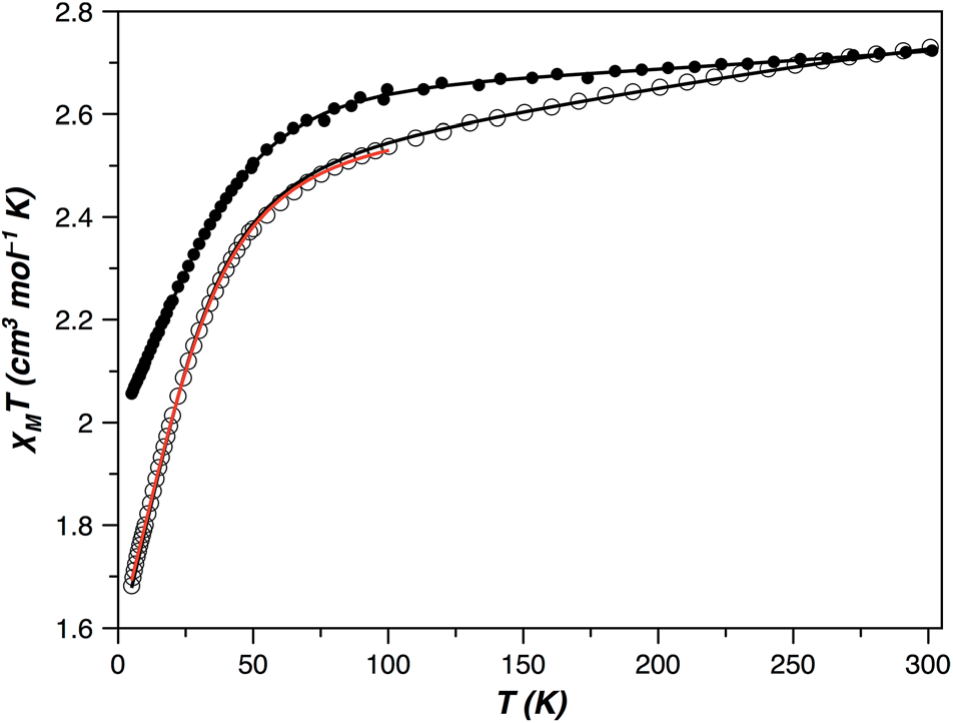
Temperature dependence of the product of the direct current (dc) molar magnetic susceptibility and the temperature (*χ*_M_ *T*) of **1** () and **2** (○). The solid lines are the best-fit curves (see text): (**1**) *g*_⊥_ = 2.11; *g*_∥_ = 2.67, |*D*| = 50.6 cm^–1^, TIP = 529 × 10^–6^ cm^3^ mol^–1^; and (**2**) *g*_⊥_ = 2.41; *g*_∥_ = 2.07, |*D*| = 35.8 cm^–1^, TIP = 437 × 10^–6^ cm^3^ mol^–1^, in black, and *α* = 1.32; *λ* = –118 cm^–1^, *Δ* = +805, TIP = 246 × 10^–6^ cm^3^ mol^–1^, in red under the SOC framework.

The direct current (dc) magnetic susceptibility behaviour of **1** and **2** in the form of *χ*_M_ *T versus T* and *M versus H*/*T* plots (*χ*_M_ and *M* being the molar magnetic susceptibility and magnetisation, and *T* and *H* the absolute temperature and the applied magnetic field) revealed the occurrence of large single-ion magnetic anisotropies (Fig. 7 and S6, ESI†). The values of *χ*_M_ *T* at room temperature were 2.72 (**1**) and 2.73 cm^3^ mol^–1^ K (**2**). They are higher than those expected for an isotropic *S* = 3/2 spin moment [*χ*_M_ *T* = 1.875 cm^3^ mol^–1^ K with *g* = 2.0] but in agreement with those expected for one high-spin d^7^ Co^II^ ion with unquenched orbital momentum. Upon cooling, *χ*_M_ *T* continuously decreases for both compounds to reach 2.06 (**1**) and 1.68 cm^3^ mol^–1^ K (**2**) at 5 K, values which suggest a different magnitude of the zfs in them. Isothermal magnetization curves in the form *M versus H*/*T* do not superimpose when a zfs or weak magnetic couplings are present, which occurred in **1** and **2** (Fig. S6, ESI†). However, if the zfs is very large, the two split Kramers doublets are well separated in energy, and a small or no temperature influence on the *M vs. H*/*T* magnetization isotherms is to be expected. In such a case, it is not possible to accurately extract the values of the zfs parameters from the magnetization curves, but the experiment allowed us to conclude that the zfs is significant in **1** and **2** and probably greater in the former compound.

The magnetic behaviour of OC-6 cobalt(ii) complexes is usually induced by a first order SOC. Consequently, the use of the SOC Hamiltonian introduced through T-P isomorphism [eqn (S1) and (S2), ESI†] is the most appropriate option in the analysis of their magnetic properties. The *α*, *λ*, *Δ* and TIP parameters in this Hamiltonian are the orbital reduction factor, spin–orbit constant, energy level splitting of the orbital moment through the lowering of the molecular symmetry and temperature-independent paramagnetism, respectively.^50^ The least-squares fit of the magnetic susceptibility data of **2** from 5 to 300 K is: *α* = 1.32, *λ* = –118 cm^–1^, *Δ* = +805 cm^–1^, TIP = 246 × 10^–6^ cm^3^ mol^–1^ and *F* = 1.2 × 10^–5^ (*F* is the agreement factor defined as ∑[*P*_exp_ – *P*_calcd_]^2^/∑[*P*_exp_]^2^, with *P* being the physical property under study). Among the six Kramers doublets coming from the coupling between the spin (*S* = 3/2) and orbit (*L* = 1) momenta in a distorted octahedron, the two lowest doublets are usually the unique populated states below 50 K; therefore, a phenomenological approach based on the zfs of an *S* = 3/2 state that considers only these doublets [as summarized in the spin Hamiltonian of eqn (S2) (ESI†)] is also justified at low temperatures. Above 50 K, a TIP term, however, should be introduced into this picture to take into account the depopulation of the remaining higher energy Kramers doublets. This approach including both terms is always efficient under 100 K but only occasionally up to room temperature. The experimental magnetic susceptibility of **2**, in the temperature range 5–100 K, was analyzed with the VPMAG program.^51^ The least-squares fit gave: *g*_⊥_ = 2.41, *g*_∥_ = 2.07, *D* = +35.8 cm^–1^ and TIP = 4.37 × 10^–6^ cm^3^ mol^–1^ with *F* = 3.0 × 10^–6^. Although usually the thermal dependence of the magnetic susceptibility cannot unambiguously determine the sign of the *D* parameter, this was possible in **1** – and also in **2** – because of the large anisotropy in the *g*-factors. Both methods, T-P isomorphism and zfs, were appropriate and provided the same energy gap between the two lowest Kramers doublets (71.6 cm^–1^, see the Magnetic measurements section in the ESI†). It is remarkable that the axial and rhombic zfs parameters and the components of the Landé *g*-tensor obtained from magnetic susceptibility and HFEPR spectroscopy are similar to the theoretical ones [*D* = +27.6 cm^–1^, *E*/*D* = 0.198, *g*_⊥_ = 2.42 (*g*_*x*_ = 2.50 and *g*_*y*_ = 2.34) and *g*_∥_ = 2.12].

The electronic structure in T-4 Co(ii) complexes is different than it is in those of OC-6 because the ground state is a non-degenerate orbital term and the SOC Hamiltonian in eqn (S1) (ESI†) is inadequate. Hence, the zfs comes from a 2^nd^ order SOC and, as a result, it is generally smaller than in the OC-6 complexes. However, geometrical distortions can transform the ground state into a magnetic degenerated or quasi-degenerated orbital term increasing the SOC contributions to the zfs. In this sense, the magnetic susceptibility data of **1** from 5 to 300 K were perfectly simulated (*F* = 2.9 × 10^–6^) through the spin Hamiltonian described by eqn (S2)† and with the following best-fit parameters: *g*_⊥_ = 2.11, *g*_∥_ = 2.67, *D* = –50.6 cm^–1^, and TIP = 529 × 10^–6^ cm^3^ mol^–1^ (Fig. 7). Once more, they are similar to those derived through NEVPT2 calculations [*D* = –57.0 cm^–1^, *E*/*D* = 0.004, *g*_⊥_ = 2.10 (*g*_*x*_ = 2.10 and *g*_*y*_ = 2.10) and *g*_∥_ = 2.77].

### Electronic structure

In ideal TBP and SQP geometries, the ground state (GS) has no angular momentum. In such cases, only a zfs coming from a 2^nd^ order SOC and modest *D* values should be expected. Large distortions of these ideal geometries could change the scene and the first excited states should come very near to the GS. The set of these excited states and the GS could be seen as a ground term with an orbital momentum, as occurred in the d^7^ high-spin OC-6 complexes. The distorted geometry of **2** leads to this situation: the GS and the two (Q_1_ and Q_2_) first excited states are quasi-degenerate (Fig. S7, ESI†) and constitute the ^4^P orbital triplet term in the higher symmetry of an ideal octahedron; therefore, the contributions from these two excited states (*D*_Q_1__ and *D*_Q_2__) can be seen as a result of a strong first order SOC. The other quartet and doublet spin states resulting from the interchange of one electron between the t_2g_ and e_g_ orbitals are moved considerably away from the ground state (GS) and they weakly contribute to the *D* parameter through a second order SOC. In consequence, the contributions to the axial zfs from the quartet spin states (*D*_Q_) in **2** – an intermediate point between the TBP and SQP geometries – are much larger than those from the doublet ones (*D*_D_) and these significant contributions are mainly sustained by the two lowest quartet excited states (Table S4, ESI†).

A first order SOC cannot occur in an ideal T-4 environment because the ground state corresponds to a singlet orbital term. In such cases, the axial zfs is smaller than in the previous geometries. Some geometrical changes, however, such as a plier distortion, can convert the GS into a doublet orbital term, significantly increasing the axial zfs by means of a 1^st^ order SOC (Fig. S7, ESI†). This is the case for **1** where the first excited quartet state (Q_1_) is close to the GS (1400 cm^–1^) and it is almost solely responsible for the very large negative axial zfs (Table S4, ESI†).

Despite **1** being distorted, the orientation of its zfs tensor is similar to that shown for the molecular axis derived from the d orbitals in an ideal T-4 symmetry (Fig. S8, ESI†). This is not the case for **2** which is highly distorted, and there is no coincidence even with the orientation observed in **1** (Fig. S8, ESI†). This fact should be the cause of the very different axial and rhombic zfs of **1** and **2**.

### Dynamic (ac) magnetic properties

**Fig. 8:**
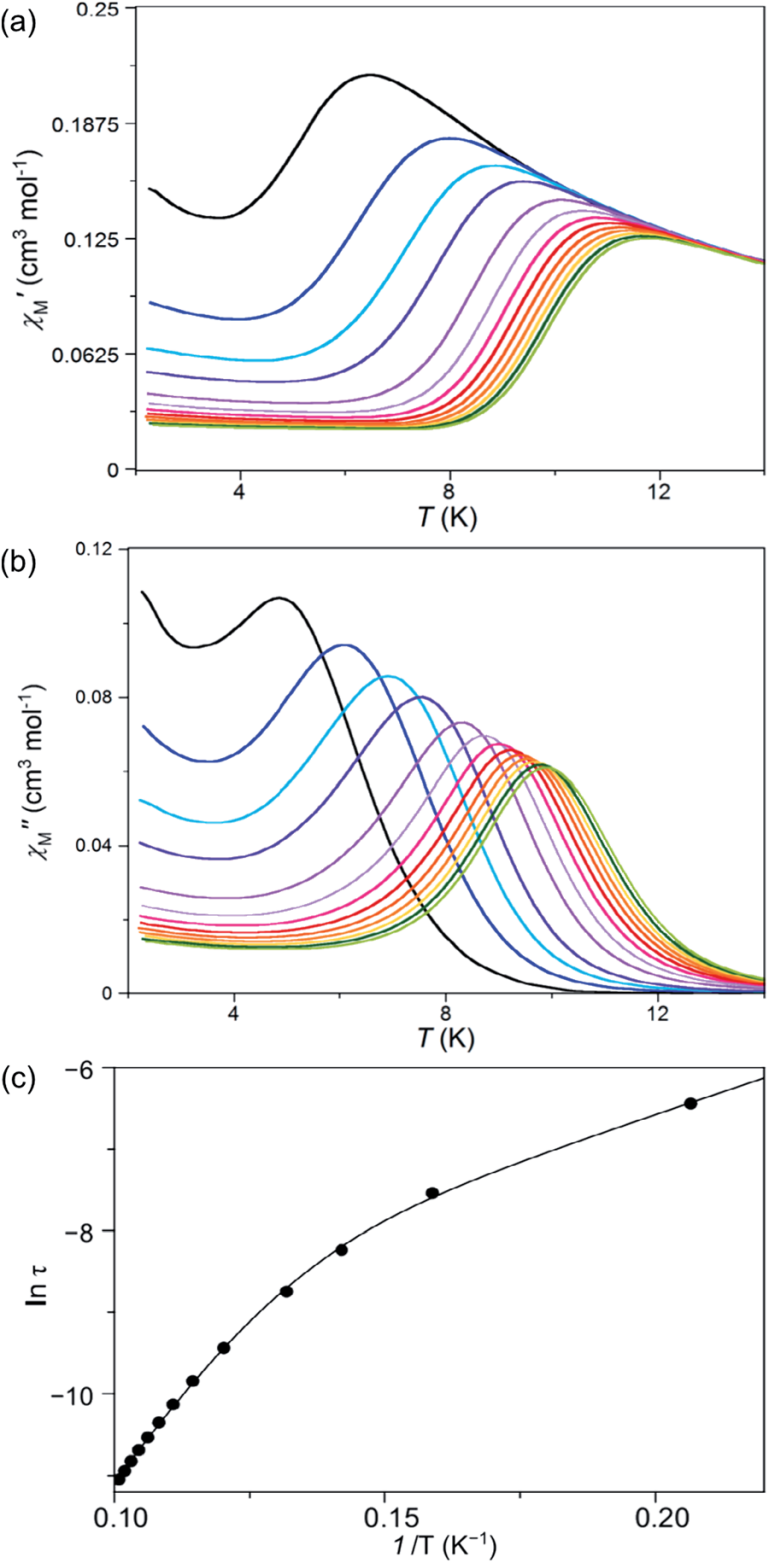
Temperature dependence of the alternating current (a) in- and (b) out-of-phase molar magnetic susceptibilities. (c) Arrhenius plot in the high temperature region of **1** under an applied static field of 1000 G with a ±5.0 G oscillating field in the frequency range of 100–10 000 Hz (from black to green). The solid line in (c) is the best-fit curve through a model with two Orbach relaxation processes (Table S5†).

Measurements of the alternating current (ac) magnetic susceptibility applying a dc magnetic field (0.0–2.5 kG) were carried out for **1** and **2**. Whereas slow magnetic relaxation effects, typical of Co(ii) SIMs, were observed for **1**, this was not the case for **2** as shown by the thermal dependence of the in-phase (*χ*′_M_) and out-phase (*χ*′′_M_) magnetic susceptibilities (Fig. 8, S9 and S10, ESI†). A blocking of the magnetization was observed for **1** below 10 K even in the absence of a dc magnetic field; however, the maxima of *χ*′′_M_ are not well defined probably because of the presence of fast quantum tunnelling relaxation even at 1000 G. In turn, compound **2** shows no maxima over 2.0 K and only incipient and very weak out-of-phase ac signals near this temperature can be observed (Fig. S9†).

Deconvolution of the thermal dependence of *χ*′′_M_ allowed us to accurately calculate the maxima of *χ*′′_M_ at a given frequency (*τ* = 1/2π*ν*, *τ* being the relaxation time). The plot of ln(*τ*) *vs.* 1/*T* follows an Arrhenius law characteristic of a thermally activated mechanism with two different relaxation processes [*τ* = {(1/*τ*_01_) exp(*E*_a1_/*k*_B_ *T*) + (1/*τ*_02_) exp(*E*_a2_/*k*_B_ *T*)}^–1^] (solid lines in Fig. 8c and S11, ESI†). Each one of these two relaxation processes is predominant in different temperature regions; *i.e.* one of them relaxes faster in a particular 1/*T* region although both coexist at any temperature. What is responsible for the curvature in the Arrhenius plot is that there is a temperature region where both processes are competing without there being a clear winner. The data processing with both processes rivalling is adequate; however, to individually analyse each area using only one relaxation process is not acceptable because the presence of another relaxation misrepresents the predominant relaxation process. The calculated values of the first pre-exponential factor (*τ*_0_) and activation energy (*E*_a_) (Table S5, ESI†) obtained this way are consistent with those found for other previously reported SIMs of OC-6 cobalt(ii) complexes.^52^ Lower values of *E*_a_ were, however, found for the second relaxation process such as is observed for other Co^II^ systems showing a double relaxation process.^49^ Like in that paper, analysis considering quantum tunnelling or Raman mechanisms for the second and faster relaxation processes was ambiguous, leading to disparate and even inconsistent values for the parameter that determines the direct, quantum tunnelling, and Raman mechanisms (see the Magnetic measurements section in the ESI†). A model with two Orbach processes, which could be related to parallel and transversal relaxation times, seems to account for the magnetic behaviour observed in **1**.

Nevertheless, against the double relaxation process, small values of the *α* parameter were extracted from almost perfect semicircle Cole–Cole plots (Table S5 and Fig. S12, ESI†), discarding the spin-glass behaviour (*α* ≥ 0.4). This fact allowed us to conclude that two observed relaxation processes occur in a unique cobalt(ii) complex. Similarly to the majority of cobalt(ii) complexes with first order SOC, the energy barrier calculated from the experimental *D* value [*E*_a_ = –*D*(*S* ^2^ – 1/4) = –2*D* = 101.2 cm^–1^] is much larger than the experimental one derived from the Arrhenius behaviour.^52^

In summary, structural changes in **1** and **2**, associated with a reversible dehydration/rehydration process, are responsible for the reversal of the sign of *D* and the disappearance of the SIM behaviour. It was originally believed that only negative *D* values permitted slow magnetic relaxation effects and such effects would not be possible for positive *D* values.^53^ The dynamic magnetic behaviours exhibited by **1** and **2** match this logical assumption. However, since about a dozen examples of Co(ii) SIMs with large and positive values of *D* can already be found in the literature, explaining the magnetic switch between **1** and **2** taking into account only the *D* sign is naïve.^17,18,23,49,54,55^ On the other hand, the magnetic relaxation through a high energy barrier induced by an axial zfs could be assisted by extra energy investment from phonons emitted by the solid network from a vibrational relaxation.^54^ Hence, the synthesis and investigation of novel cobalt(ii) complexes are essential to understanding this singular magnetic behaviour, although an improvement of the current theoretical models or the development of new ones that answer the open questions will be a landmark in the field.

## Conclusions

We have developed a new strategy for the design and synthesis of multifunctional molecular materials showing reversible magnetic and optical switching. Our approach is based on the use of mononuclear complexes that exhibit slow magnetic relaxation behaviour and a low coordination number. This coordination environment should allow an alteration of the coordination sphere with the entry of small solvent molecules, changing the physical properties. The reversible water sorption/release process, a cornerstone of this molecular switch, is possible through the presence of a local sterically constrained environment that reduces the structural plasticity of the complexes, but without fully preventing it. This chemical change can be easily modulated through the relative humidity or the temperature of the environment. Overall, in this novel class of switchable cobalt(ii)-based SIMs, the reversible sorption behaviour coming with dramatic colour changes and the appearance or/and disappearance of the slow magnetic relaxation effects propose this system to act as a magnetic switch.
