# Supplementary material for: Reversible solvatomagnetic switching in a single-ion magnet from an entatic state
Source: Chem Sci. 2017 Feb 13;8(5):3694–702. doi: 10.1039/c6sc05188j (PMC5437488; doi:10.1039/c6sc05188j)

Electronic Supplementary Material (ESI) for Chemical Science.
This journal is © The Royal Society of Chemistry 2017

Supplementary Information (SI) for the manuscript:

Reversible solvatomagnetic switching in a single-ion magnet from an entatic state

Julia Vallejo, Emilio Pardo*, Marta Viciano-Chumillas, Isabel Castro, Pedro Amorós, Mariadel Déniz, Catalina Ruiz-Pérez, Consuelo Yuste-Vivas, J. Krzystek, Miguel Julve, Francesc Lloret & Joan Cano*

METHODS

SYNTHESES

$[Co^{II}(dmbpy)_2](ClO_4)_2$ **1** and $[Co^{II}(dmbpy)_2(H_2O)](ClO_4)_2$ **2**: An acetonitrile (**1**) and methanolic (**2**) solutions (5 cm^3) of dmbpy (0.184 g, 1.0 mmol) were added dropwise to solutions of $Co(ClO_4)_2 \cdot 6H_2O$ (0.187 g, 0.5 mmol) in acetonitrile (10 cm^3) under continuous stirring. The resulting deep red (**1**) and pale orange (**2**) solutions were filtered off. Resulting solutions were layered with a diethyl ether solution on an essay tube (**1**) or allowed to evaporate at room temperature (**2**). X-ray quality dark red (**1**) and bright orange prisms (**2**) appeared after a few hours on standing at room temperature [0.291 g, 92% yield (**1**) and 0.250 g, 77% yield (**2**)]. Anal. Calcd. for $C_{24}H_{24}Cl_2CoN_4O_8$ (626.30), **1**: C, 46.02; H, 3.86; N, 8.95%. Found: C, 45.76; H, 3.91; N, 8.92%. Anal. Calcd. for $C_{24}H_{26}Cl_2CoN_4O_9$ (644.32), **2**: C, 44.74; H, 4.07; N, 8.70%. Found: C, 45.01; H, 3.98; N, 8.75%. UV–Vis (CH_3CN) for **1**: λ_{max} (ε) = 239 (501700), 292 (723230), 542 (sh) (102), 572 nm (189 M^{-1} cm^{-1}); and for **2**: λ_{max} (ε) = 237 (327740), 291 (502230), 527 (12), 569 (sh) nm (9 M^{-1} cm^{-1}).

SINGLE CRYSTAL X-RAY DIFFRACTION

X-ray diffraction data on single crystals of **1** and **2** were collected at 293(2) K on a Bruker Apex II (**1**) and Bruker-Nonius KappaCCD (**2**) diffractometer by using graphite-monochromated Mo-Kα radiation (λ = 0.71073 Å). All calculations for data reduction, structure solution, and refinement were done through the *SAINT* and *SADABS* programmes[1,2]. The structures of **1** and **2** were solved by direct methods and subsequently completed by Fourier recycling using the *SHELXTL* software package[3]. All non-hydrogen atoms were refined anisotropically. The hydrogen atoms from the dmbpy ligands of **1** and **2** were set in calculated positions and refined with a riding model, while those of the

coordinated water molecule of **2** were located from Fourier differences and refined with isotropic thermal factors. One of the perchlorate anions of **1** is found to be disordered, so we have modelised it with one of the oxygen atoms occupying two different positions with an occupancy ratio of 0.66:0.34. The final geometrical calculations and the graphical manipulations were carried out with *PLATON* and *CRYSTALMAKER* programmes[4,5]. Crystallographic data (excluding structure factors) for the structures reported in this paper have been deposited with the Cambridge Crystallographic Data Centre as supplementary publication no. CCDC–952077 (**1**) and 938463 (**2**). Copies of the data can be obtained free of charge on application to CCDC, 12 Union Road, Cambridge CB21EZ, UK (fax: (+44) 1223-336-033; e-mail: deposit@ccdc.cam.ac.uk).

X-RAY POWDER DIFFRACTION

X-ray powder diffraction (XRPD) patterns of crystalline powdered samples of **1** and **2** were collected at room temperature on a D8 Avance A25 Bruker diffractometer by using graphite-monochromated Cu-Kα radiation (λ = 1.54056 Å). XRPD measurements of the moisture-sensitive dehydrated derivative were carried out by using an Anton Paar chamber working at 20 ºC under a controlled relative humidity of 12%. The XRPD patterns were recorded by using a LYNXEYE XE 1-dimensional detector for ultra fast X-ray diffraction measurements. Spectra were recorded in a continuous way every two minutes over the angular range of $14 \leq 2\theta \leq 16^{\circ}$, where there are clear differences in the peak positions of the XRPD patterns of **1** and **2**.

THERMOGRAVIMETRIC STUDY

The thermogravimetric analysis (TGA) was performed on a very small amount (2 mg) of crystalline powdered samples of **2** under a dry N_2 atmosphere with a Mettler Toledo TGA/STDA 851^{e} thermobalance operating at a heating rate of 10 ºC min^{-1}.

VAPOUR SORPTION STUDIES

Equilibrium vapour water sorption measurements were performed at 25 ºC on crystalline powdered samples of **1** using a conventional gravimetric instrument. Samples were dried under *RH* = 0 prior to their analysis, and they were then treated at different *RH* values up to constant weight.

OPTICAL MICROSCOPY

Optical microscopy zoom images (80x) of single crystals and crystalline powdered samples of **1** and **2** were recorded using a Nikon SMZ1000 optical microscope equipped with a Nikon Digital Sight DS-Fi1 camera.

SPECTROSCOPIC MEASUREMENTS

UV–Vis spectra of crystalline powdered samples of **1** and **2** were recorded at room temperature on a JASCO V-670 spectrophotometer. X-band EPR spectra (ν = 9.47 GHz) of crystalline powdered samples of **1** and **2** were recorded in non-saturating conditions at 4.0 K on a Bruker ER 200 D spectrometer equipped with a helium cryostat. HFEPR spectra of **1** and **2** were recorded at 4.5 K on polycrystalline samples (20-25 mg) by using a homodyne spectrometer associated with a 15/17-T superconducting magnet and a frequency range from 52 to 610 GHz. Detection was provided with an InSb hot electron bolometer (QMC Ltd., Cardiff, UK). The magnetic field was modulated at 50 kHz for detection purposes. A Stanford Research Systems SR830 lock-in amplifier converted the modulated signal to dc voltage. The single-frequency spectra were simulated with the SPIN software.

MAGNETIC MEASUREMENTS

Static direct current (dc) measurements were carried out on 40 mg of **1** and **2** by powdering and restraining the samples in order to prevent any displacement due to the magnetic anisotropy. Variable-temperature (2.0–300 K) dc magnetic susceptibility under

an applied field of 0.25 ($T < 20$ K) and 5.0 kG ($T \geq 20$ K), and variable-field (0–7.0 T) magnetisation in the temperature range from 2 to 10 K were recorded with a Quantum Design SQUID magnetometer. Variable-temperature (2.0–10 K) alternating current (ac) magnetic susceptibility measurements under ±5.0 G oscillating field at frequencies in the range of 0.1–10 kHz were carried out on frozen diethyl ether solutions of crystalline samples under different applied static dc fields in the range 0.0–2.5 kG with a Quantum Design Physical Property Measurement System (PPMS). The magnetic susceptibility data were corrected for the diamagnetism of the constituent atoms and the sample holder.

The energy gaps between the two lowest Kramers doublets in **1** and **2** were extracted from diagonalizations of the energy matrix built with the values of α, λ and Δ parameters obtained from the analysis of the experimental thermal dependence of the magnetic susceptibility. Similar energy gap [or the activation energy (E_a)] for the phenomenological approach based on a *zfs* of a S = 3/2 state was evaluated from the equation $E_a = 2\sqrt{D^2 + 3E}$, the E value being always positive.

Several relaxation mechanisms could be responsible for the slow magnetic relaxation observed in **1**. Therefore, different models were evaluated:

A model with direct and Orbach relaxation mechanism was tested, and the simulations were only moderately good with the highest applied magnetic field. A model accounting for Orbach plus QTM processes was also used. The results were just modest, particularly for the data using the lowest magnetic field. Moreover, the dependence on the magnetic field of the coefficient that controls the QTM process and the activation energy in the Orbach mechanism was not as expected. Finally, a model taking into account a Raman relaxation process in addition to the Orbach plus QTM processes, matched up perfectly the experimental data in the full range of the applied magnetic fields. However, an overparameterization could be at the origin of this perfect fit and, indeed, the obtained

values confirm this suspicion: Firstly, the dependence on the magnetic field of the parameter that controls the QTM process utterly lacks of consistency and, secondly, the exponent for the temperature in the Raman term is strongly dependent on the magnetic field, particularly at low magnetic fields. Overall, the problems found with these analyses together with the good and consistent results obtained using a relaxation mechanism with two Orbach processes (even if some small discrepancies are also found at lower magnetic field due probably to the poorer measurements and the more effective role of the quantum tunneling mechanism) led us to reject the inclusion of the direct, Raman or QTM processes together the Orbach relaxation.

COMPUTATIONAL DETAILS

Complete active space (CAS) calculations and a second order N-electron valence state perturbation applied on their wave functions were performed on the structurally characterized molecular geometries of **1** and **2** with the version 3.0 of the ORCA programme using the *TZVP* basis set proposed by Ahlrichs and the auxiliary TZV/C Coulomb fitting basis sets[6–12]. The contributions from 10 quartet and 20 doublet excited states generated from an active space with seven electrons in five d orbitals have been included, the quartet states showing different energies due to the rhombic plier distortion on the TD (**1**) or the rhombic distortion on the TBP (**2**) geometries.

A study of the release of the coordinated water molecule from **2** to transform into **1** was done in gas phase with the Gaussian09 package using the CAM-B3LYP functional (a long range corrected version of B3LYP) and the quadratic convergence approach[13–17]. The Ahlrichs double-ζ basis set was used for all atoms[18]. All molecular geometries were optimized and verified by means of a vibrational frequency calculation. The geometry of the transition state for the dehydration process was also optimized by using the Berny

algorithm. Free energies at 298 K were evaluated from the electronic, vibrational, rotational and translational contributions.

Electronic excitations on full experimental geometries of **1** and **2** were calculated through the time-dependent DFT formalism (TDDFT) with the Gaussian 09 program[19,20]. A polarizable continuum model (PCM) was introduced in the calculations with the parameters corresponding to the acetonitrile.[21] Triple- and double- ζ all electron basis sets, as proposed by Ahlrichs *et al.*[7,18], were used for the metal ions and for the rest of atoms, respectively. A CAM-B3LYP functional (a long range corrected version of B3LYP) was used in this study.

1. SAINT, version 6.45; Bruker Analytical X-ray Systems; Madison, WI. (2003).

2. Sheldrick, G. M. SADABS Program for absorption correction, version 2.10; Analytical X-ray Systems; Madison,WI. (2003).

3. Sheldrick, G. M. A short history of SHELX. *Acta Crystallogr. A.* **64,** 112–122 (2008).

4. Spek, A. L. Structure validation in chemical crystallography. *Acta Crystallogr. Sect. D Biol. Crystallogr.* **65,** 148–155 (2009).

5. Palmer, D. CrystalMaker. (1996).

6. Neese, F. The ORCA program system. *Wires Comput Mol Sci* **2,** 73–78 (2012).

7. Schafer, A., Huber, C. & Ahlrichs, R. Fully Optimized Contracted Gaussian-Basis Sets of Triple Zeta Valence Quality for Atoms Li to Kr. *J. Chem. Phys.* **100,** 5829–5835 (1994).

8. Eichkorn, K., Treutler, O., Öhm, H., Häser, M. & Ahlrichs, R. Auxiliary basis sets to approximate Coulomb potentials (Chem. Phys. Letters 240 (1995) 283-290). *Chem. Phys. Lett.* **242,** 652–660 (1995).

9. Eichkorn, K., Weigend, F., Treutler, O. & Ahlrichs, R. Auxiliary basis sets for main row atoms and transition metals and their use to approximate Coulomb potentials. *Theor Chem Acc* **97,** 119–124 (1997).

10. Angeli, C., Cimiraglia, R., Evangelisti, S., Leininger, T. & Malrieu, J.-P. Introduction of n-electron valence states for multireference perturbation theory. *J. Chem. Phys.* **114,** 10252 (2001).

11. Angeli, C., Cimiraglia, R. & Malrieu, J.-P. N-electron valence state perturbation theory: A spinless formulation and an efficient implementation of the strongly

contracted and of the partially contracted variants. *J. Chem. Phys.* **117,** 9138 (2002).

12. Angeli, C., Cimiraglia, R. & Malrieu, J.-P. N-electron valence state perturbation theory: a fast implementation of the strongly contracted variant. *Chem. Phys. Lett.* **350,** 297–305 (2001).

13. Frisch, M. J. *et al.* Gaussian 09. (2009).

14. Becke, A. D. Density functional exchange energy approximation with correct asymptotic behavior. *Phys. Rev. A* **38,** 3098–3100 (1988).

15. Becke, A. D. Density functional thermochemistry. 3. The role of the exact exchange. *J Chem Phys* **98,** 5648–5652 (1993).

16. Lee, C. T., Yang, W. T. & Parr, R. G. Development of the Colle-Salvetti correlation energy formula into a functional of the electron density. *Phys Rev B* **37,** 785–789 (1988).

17. Yanai, T., Tew, D. P. & Handy, N. C. A new hybrid exchange–correlation functional using the Coulomb-attenuating method (CAM-B3LYP). *Chem. Phys. Lett.* **393,** 51–57 (2004).

18. Schäfer, A., Horn, H. & Ahlrichs, R. Fully optimized contracted Gaussian basis sets for atoms Li to Kr. *J. Chem. Phys.* **97,** 2571 (1992).

19. Casida, M. E., Jamorski, C., Casida, K. C. & Salahub, D. R. Molecular excitation energies to high-lying bound states from time-dependent density-functional response theory: Characterization and correction of the time-dependent local density approximation ionization threshold. *J Chem Phys* **108,** 4439–4449 (1998).

20. Casida, M. E. & Huix-Rotllant, M. Progress in Time-Dependent Density-Functional Theory. *Annu. Rev. Phys. Chem. Vol 63* **63,** 287–323 (2012).

21. Tomasi, J., Mennucci, B. & Cammi, R. Quantum mechanical continuum solvation models. *Chem. Rev.* (2005). doi:10.1021/cr9904009

Table S1 Summary of crystallographic data for 1 and 2

	**1**	**2**
Formula	$C_{24}H_{24}Cl_2CoN_4O_8$	$C_{24}H_{26}Cl_2CoN_4O_9$
M (g mol^{-1})	626.30	644.32
Crystal system	monoclinic	monoclinic
Space group	$P2_1/c$	$P2_1/c$
a (Å)	17.694(5)	18.258(3)
b (Å)	11.623(7)	11.0640(9)
c (Å)	13.491(7)	14.3340(13)
β (°)	103.852(5)	101.816(5)
V (Å^3)	2693.8(17)	2834.3(6)
Z	4	4
ρ_{calc} (g cm^{-3})	1.544	1.510
μ (mm^{-1})	7.302	0.851
T (K)	200(2)	293(2)
F(000)	1284	1324
Collcd. reflections	14642	22121
Indep. reflections (R_{int})	5471 (0.0732)	6756 (0.0765)
Obs. reflections [$I > 2\sigma(I)$]	3014	3806
Data / restraints / parameters	5257 / 0 / 353	6756 / 2 / 373
R_1^a [$I > 2\sigma(I)$] (all)	0.0921 (0.1410)	0.0786 (0.1511)
wR_2^b [$I > 2\sigma(I)$] (all)	0.2395 (0.2735)	0.1680 (0.2024)
Goodness-of-fit	1.070	1.066
S^c	1.070	1.066

^{a} $R_1 = \sum(|F_o| - |F_c|)/\sum|F_o|$. ^{b} $wR_2 = [\sum w(F_o^2 - F_c^2)^2/\sum w(F_o^2)^2]^{1/2}$. ^{c} $S = [\sum w(|F_o| - |F_c|)^2/(N_o - N_p)]^{1/2}$.

Table S2 Selected bond distances (Å) and interbond angles (°) for 1 and 2[a]

	**1**	**2**
Co(1)–N(1)	1.981(6)	2.085(4)
Co(1)–N(2)	2.002(6)	2.088(4)
Co(1)–N(3)	2.014(6)	2.074(4)
Co(1)–N(4)	2.004(6)	2.110(4)
Co(1)–O(1w)		2.079(4)
N(1)–Co(1)–N(2)	83.5(2)	80.00(15)
N(1)–Co(1)–N(3)	123.2(2)	104.97(15)
N(1)–Co(1)–N(4)	131.4(2)	104.39(15)
N(2)–Co(1)–N(3)	115.7(2)	113.06(16)
N(2)–Co(1)–N(4)	124.3(2)	165.82(16)
N(3)–Co(1)–N(4)	83.0(2)	79.18(18)
O(1w)–Co(1)–N(1)		139.42(17)
O(1w)–Co(1)–N(2)		89.15(16)
O(1w)–Co(1)–N(3)		115.21(18)
O(1w)–Co(1)–N(4)		78.76(16)

[a] Estimated standard deviations are given in parentheses.

Table S3 Experimental and theoretical electronic transitions in the visible region of acetonitrile solutions of 1 and 2

	**1**		**2**	
	λ [a]	ε, f*10^4 [b]	λ [a]	ε, f*10^4 [b]
**Experimental**	514	58.2	527	14.1
	548	94.2	544	0.7
	572	126.2	573	2.4
	588	60.5		
**Theoretical**	590	15	494	5
	603	15	530	2
	871	7	876	2

[a] Wavelength in nm. [b] Molar absorption coefficient in M^{-1} cm^{-1} for the experimental spectra and oscillator strength for the theoretical electronic transitions.

Table S4 More significant contributions from the quartet (D_Q) and doublet (D_D) excited spin states to the axial *zfs* D parameter and their energies in 1 and 2 obtained from the NEVPT2 calculations. Blue grey colour is used to mark the states that, according to Figure S7, can give a quasi degenerate ground orbital term.

		**1** [a]		**2** [a]	
	**State**	D_i	**Energy**	D_i	**Energy**
**Quartet** (D_Q)		–60.0		+25.6	
	GS	+0.0	0	+0.0	0
	Q_1	–73.9	1400	+19.0	1564
	Q_2	+5.5	7933	+8.2	2366
	Q_3	+6.6	8263	+8.8	5078
	Q_4	–0.6	8763	–10.6	7264
**Doublet** (D_D)	D_1	+2.5	15679	– 0.2	9918
	D_0	–0.6	15969	–1.0	13629

[a] Contributions to D and energy in cm^{-1} and spin-spin contribution to D are shown in grey box.

Table S5 Selected ac magnetic data at different applied dc fields for 1

$H^{[a]}$ (G)	$\tau_0(1)^{[b]} \times 10^9$ (s)<br>$\tau_0(2)^{b} \times 10^5$ (s)	$E_a^{[b]}$ (cm^{-1})<br>$E_a^{[b]}$ (cm^{-1})	α[c]	$\chi_S^{[c]}$ (cm^3 mol^{-1})	$\chi_T^{[c]}$ (cm^3 mol^{-1})
250	1.79<br>17.7	63.0<br>4.68	0.121 (8 K)<br>0.109 (9 K)<br>0.113 (10 K)	0.075 (8 K)<br>0.061 (9 K)<br>0.040 (10 K)	0.198 (8 K)<br>0.176 (9 K)<br>0.159 (10 K)
500	1.25<br>2.28	66.2<br>13.1	0.084 (8 K)<br>0.095 (9 K)<br>0.096 (10 K)	0.031 (8 K)<br>0.022 (9 K)<br>0.010 (10 K)	0.198 (8 K)<br>0.176 (9 K)<br>0.157 (10 K)
1000	1.10<br>1.62	67.1<br>15.5	0.065 (8 K)<br>0.074 (9 K)<br>0.065 (10 K)	0.016 (8 K)<br>0.011 (9 K)<br>0.010 (10 K)	0.199 (8 K)<br>0.175 (9 K)<br>0.157 (10 K)
2500	0.36<br>0.74	73.9<br>19.2	0.049 (8 K)<br>0.070 (9 K)<br>0.088 (10 K)	0.013 (8 K)<br>0.008 (9 K)<br>0.001 (10 K)	0.196 (8 K)<br>0.174 (9 K)<br>0.158 (10 K)

[a] Applied dc magnetic field. [b] The values of the pre-exponential factor (τ_0) and activation energy (E_a) are calculated through the Arrhenius law [$\tau = \{(1/\tau_{01}) \exp(E_{a1}/k_BT) + (1/\tau_{02}) \exp(E_{a2}/k_BT)\}^{-1}$]. [c] The values of the α parameter, adiabatic (χ_S) and isothermal (χ_T) susceptibilities are calculated from the experimental data at different temperatures through the generalized Debye law (see text).

EQUATION S1:

$$H_{SOC} = \alpha\lambda\hat{L}\cdot\hat{S} + \Delta\left[\hat{L}_z^2 - L(L+1)/3\right] + \beta H\left(-\alpha\hat{L} + g_e\hat{S}\right)$$

EQUATION S2:

$$H_{zfs\,+\,zeeman} = D\left[\hat{S}_z^2 - S(S+1)/3\right] + E\left(\hat{S}_x^2 - \hat{S}_y^2\right) + \beta H\left(g_{\parallel}\hat{S}_z + g_{\perp}\left(\hat{S}_x + \hat{S}_y\right)\right)$$

FIGURE S1 Molecular and crystal structures. a, Perspective view of the π-π stacking interactions (dashed line) in **1** between the pyridyl rings from two neighbouring complexes. **b,** Perspective view of the hydrogen bonds (dashed lines) generated in the crystal structure of **2**. **c** and **d**, Views of the crystal packing of **1** and **2** along the crystallographic *c* axis. Molecular entities are shown using sticks using blue, red, yellow and grey colours for the N, O, Cl and C atoms, respectively. The hydrogen atoms are omitted for clarity and the Co and O_{wat} atoms are represented with purple and red spheres, respectively. Selected intermetallic distances (Å): Co(1)–Co(1)^{II} = 7.809(1) (**1**) and 8.6055(10) (**2**); Co(1)^{I}–Co(1)^{II} = 8.975(1) (**1**) and 10.454(2) (**2**) [symmetry code: (I) = – *x*, *y* + 1/2, – *z* + 1/2 (**1**) and – *x*, *y* + 1/2, – *z* – 1/2 (**2**); (II) = *x* + 1, – *y* + 1/2, *z* + 1/2 (**1**) and *x* + 1, – *y* + 1/2, *z* + 3/2 (**2**)].

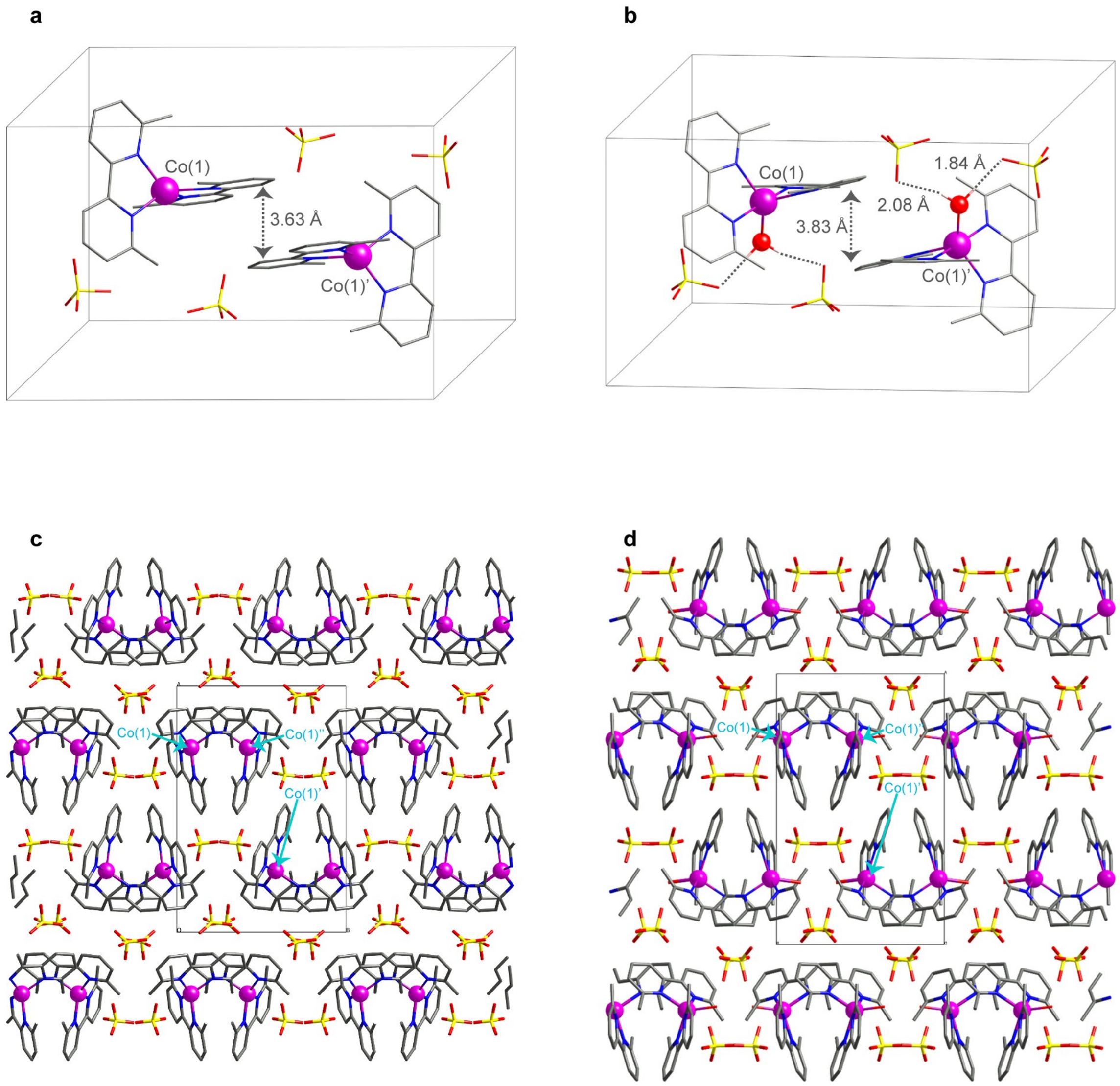
a
Co(1)
3.63 Å
Co(1)'
b
Co(1)
1.84 Å
2.08 Å
3.83 Å
Co(1)'
c
Co(1)
Co(1)'
Co(1)'
d
Co(1)
Co(1)'
Co(1)'

FIGURE S2 Thermal stability and XRPD spectra on single crystals. a, TGA of **2** under a dry N_2 atmosphere. The inset shows the release of the coordinated water molecule. **b,** XRPD patterns of the dehydrated and rehydrated derivatives (red and blue solid thin lines, respectively). The bold lines are the calculated XRPD patterns of **1** and **2**.

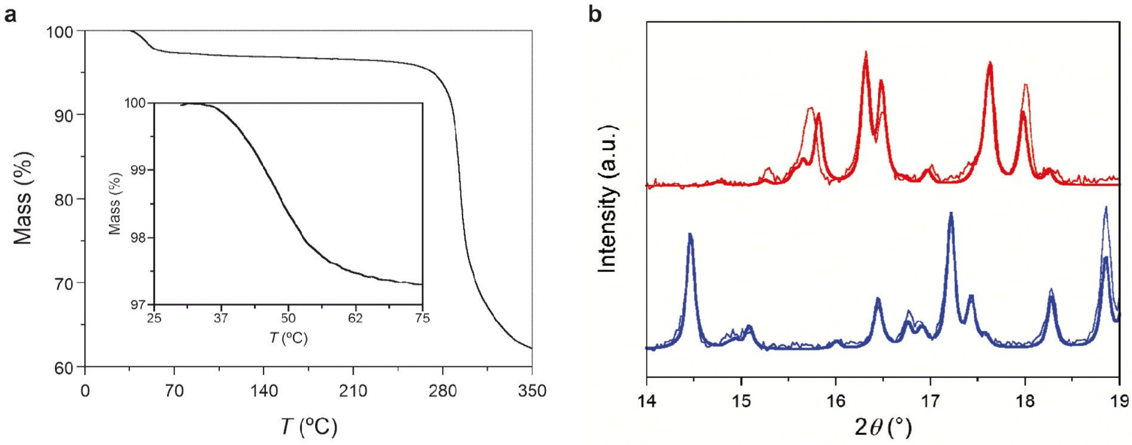

FIGURE S3 Spectroscopic properties. a and **b,** UV–Vis spectra at room temperature and X-band EPR spectra at 4.0 K of a crystalline powdered sample of **1** (red solid line) and **2** (blue solid line). The bold line in **b** corresponds to the best simulation of the EPR data of **2** with $g_1 = 2.10$, $g_2 = 4.05$, and $g_3 = 5.54$. ε and λ are the molar absorptivity and the wavelength in the UV–Vis spectra, and A_i are the components of the hyperfine coupling constant in EPR simulation.

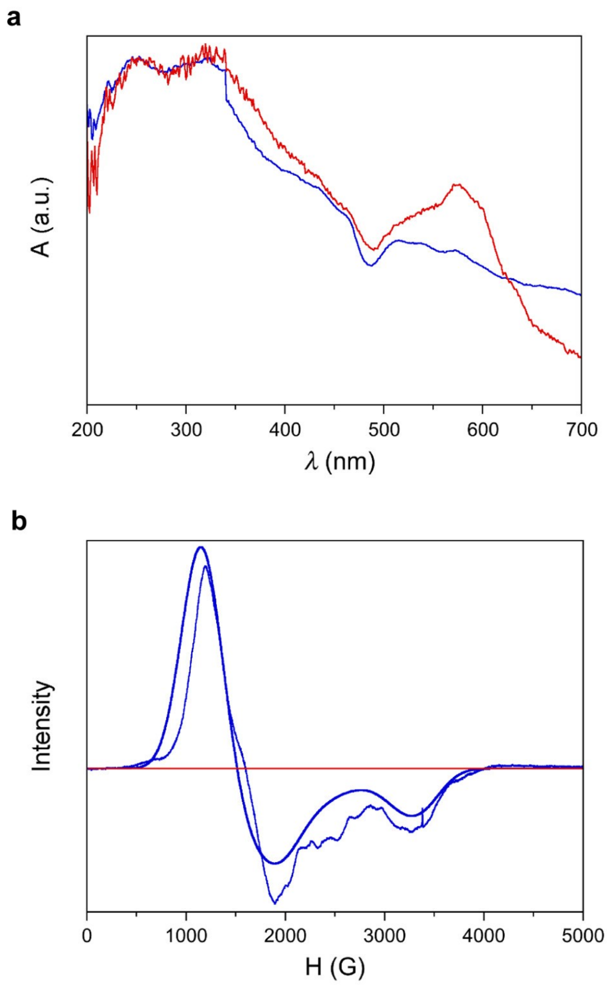

FIGURE S4. HFEPR spectrum of **2** recorded at 4.5 K and 319 GHz (black trace). Simulations using S = 3/2, $|D|$ = 28 cm^{-1} (corresponding to the result of NEVPT2 calculations), $|E/D|$ = 0.123, $g_{\perp}$ = 2.37, $g_{\parallel}$ = 2.15 are in blue and red traces for positive and negative axial D parameter.

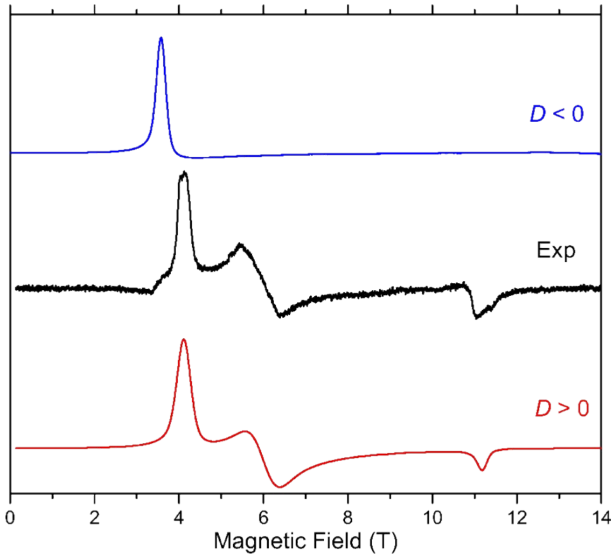

FIGURE S5 Multifrequency HFEPR study on 2. Field *vs*. frequency map of the turning points in the HFEPR spectra of **2** at 4.5 K. The squares are experimental points while the lines were simulated by using an axial *zfs* obtained by NEVPT2 ($|D|$ = 28 cm^{-1}) and best-fitted spin Hamiltonian parameters: S = 3/2; $|E/D|$ = 0.125, $g_{\perp}$ = 2.37 and $g_{\parallel}$ = 2.16. Different colors mark particular turning points: red, magnetic field B_0 parallel to the x axis of the zfs tensor; blue, $B_0 \parallel y$; black, $B_0 \parallel z$. The lines with no experimental points on them represent transitions within the excited $|S, M_S\rangle = |3/2, \pm 3/2\rangle$ Kramers doublet that is not populated at low temperatures.

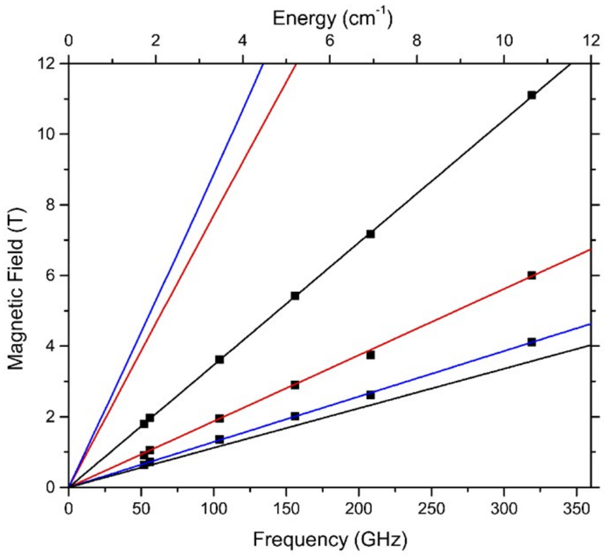

FIGURE S6 Dc magnetic properties. a and **b**, Field dependence of the magnetization (M) of **1** and **2** in the temperature range starting at 2.0 K and increasing it by one unit until 10.0 K (from black to palest grey dots). The solid lines are only eye-guides.

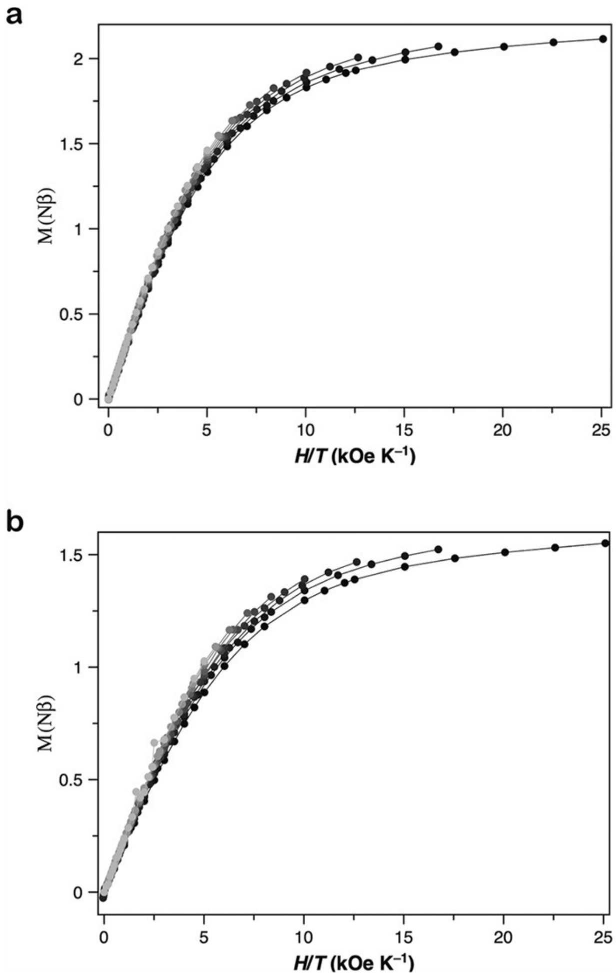

FIGURE S7 Electronic structures. Schematic splitting of the energy levels of the 3d orbitals and lowest quartet (S = 3/2) states for **1** (a) and **2** (b). Molecular orbitals were generated with a cut-off equal to 0.02 e bohr^{-3}. A high-spin Co^{II} ion (d^7) has been used to fill the 3d orbitals. The used labels correspond to the notation of the d orbital; GS and Q_i refer to the ground and the i excited quartet states. Blue grey balloons are used to point out the quasi-degenerate states that conform an orbital term.

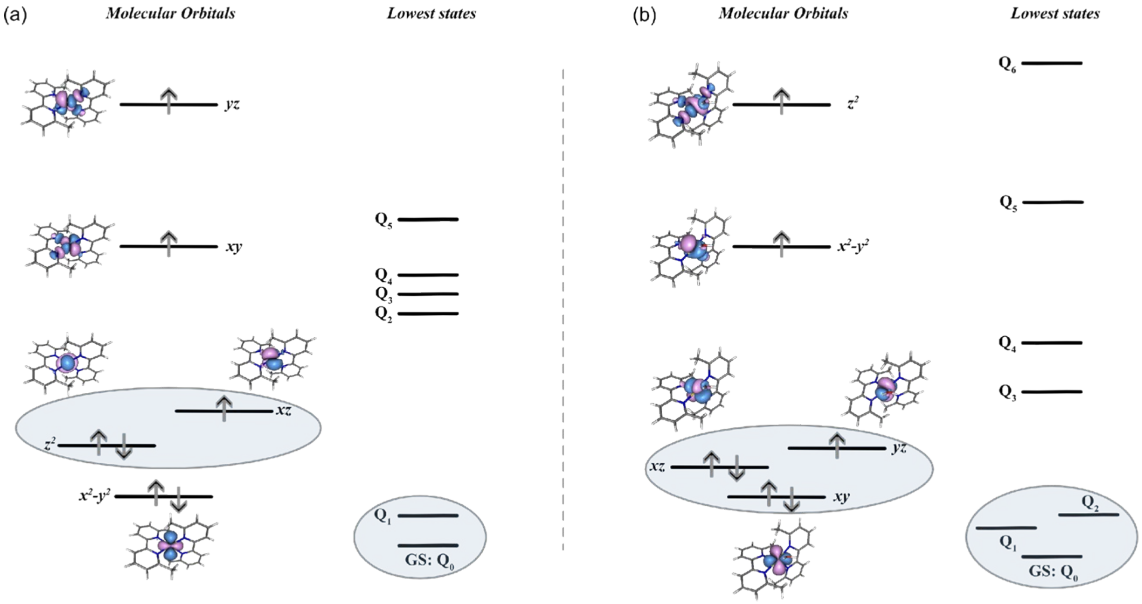

FIGURE S8. Relative orientation of the *D* tensor on Co^{II} ions in **1** (left) and **2** (right) obtained from NEVPT2 calculations. Colored sticks represent the reference axis *x* (cyan), *y* (green), and *z* (orange) of the *D* tensor. For clarity, hydrogen atoms were hidden.

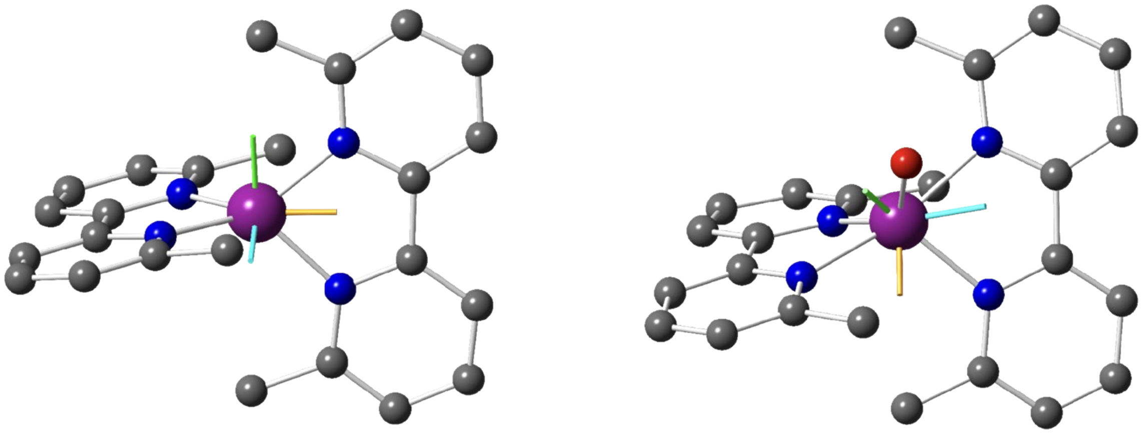

FIGURE S9. Temperature dependence of χ_M' (left) and χ_M'' (right) of **2** under applied static fields of 0 (a), 1000 (b) and 2500 G (c) with a ±5.0 G oscillating field in the frequency range of 100–10000 Hz (from black to green).

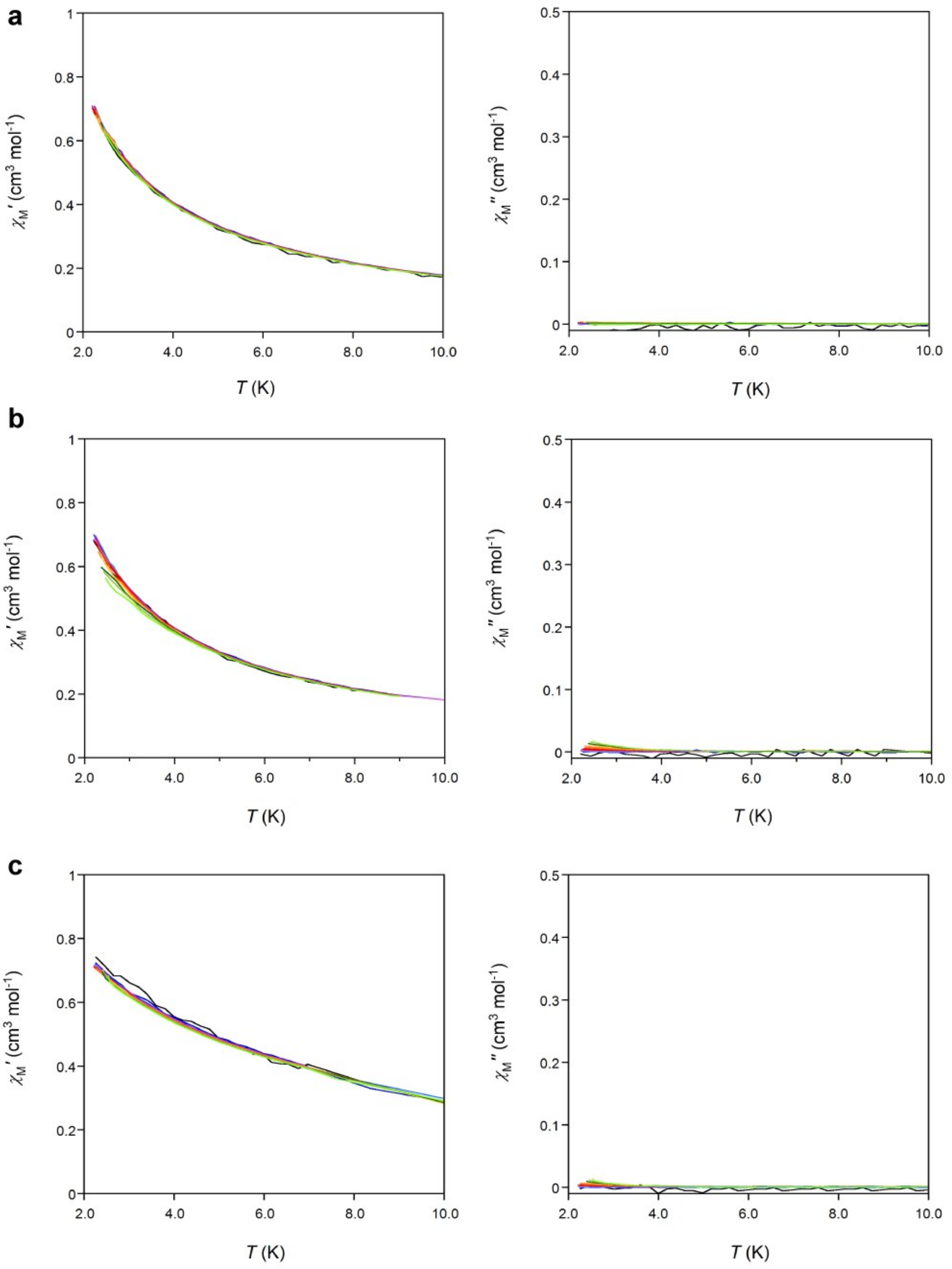

FIGURE S10. Temperature dependence of χ_M' (left) and χ_M'' (right) of **1** under applied static fields of 0 (a), 100 (b), 250 (c), 500 (d), and 2500 G (e) with a ±5.0 G oscillating field in the frequency range of 100–10000 Hz (from black to green).

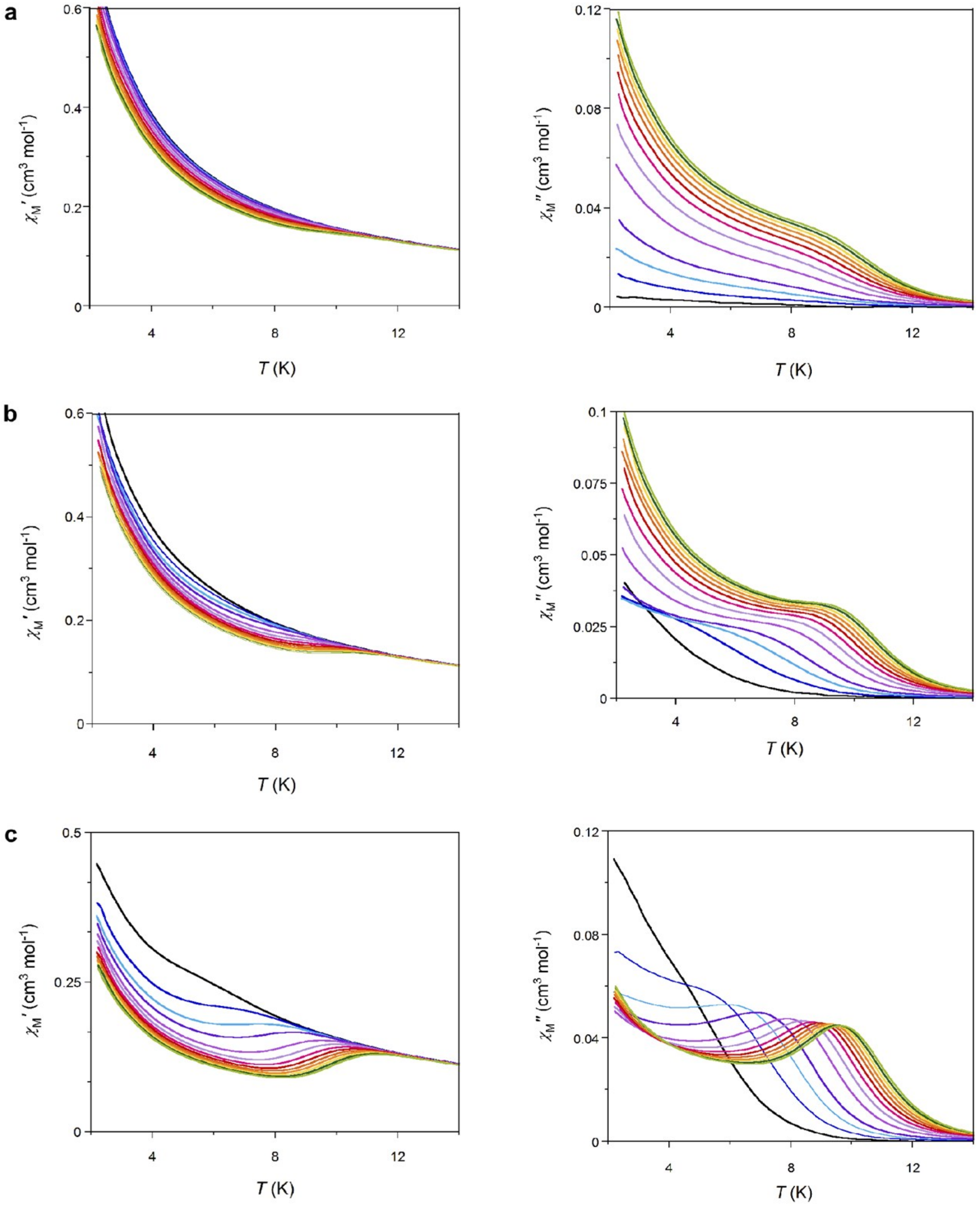

d

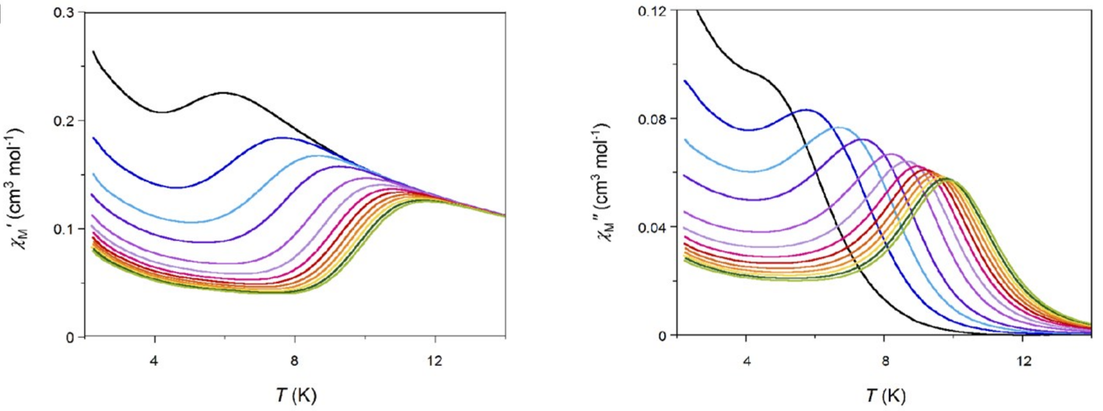
0.3
0.2
0.1
0
χ_M' (cm^3 mol^{-1})
4
8
12
T (K)
0.12
0.08
0.04
0
χ_M'' (cm^3 mol^{-1})
4
8
12
T (K)

e

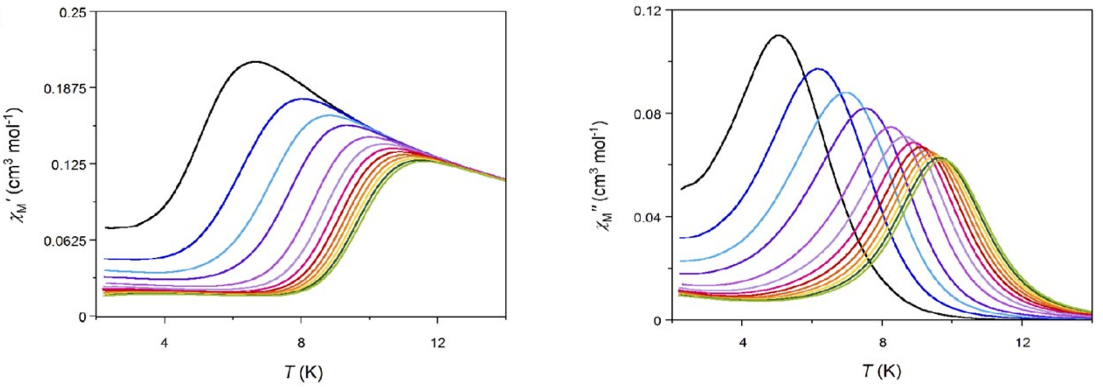
0.25
0.1875
0.125
0.0625
0
χ_M' (cm^3 mol^{-1})
4
8
12
T (K)
0.12
0.08
0.04
0
χ_M'' (cm^3 mol^{-1})
4
8
12
T (K)

FIGURE S11. Arrhenius plots in the high temperature region of **1** under applied static fields of 250 (a), 500 (b), 1000 (c), and 2500 (d). The solid lines are the best-fit curves through a model with two Orbach relaxation processes (Table S5).

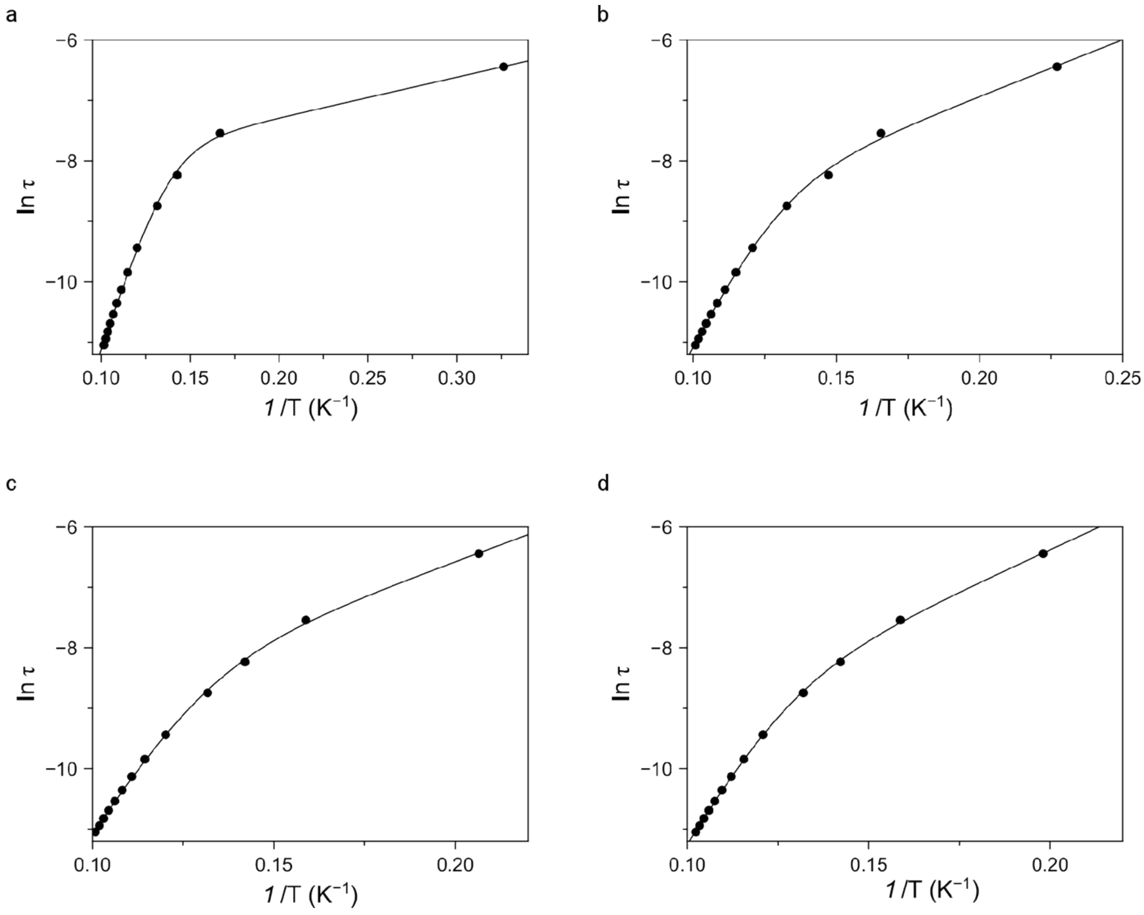

FIGURE S12. Cole-Cole plots at 8 (℘), 9 (℘), and 10 K (℘) for **1** under applied static fields of 250 (a), 500 (b), 1000 (c), and 2500 G (d). The solid lines are the best-fit curves (see Table S5).

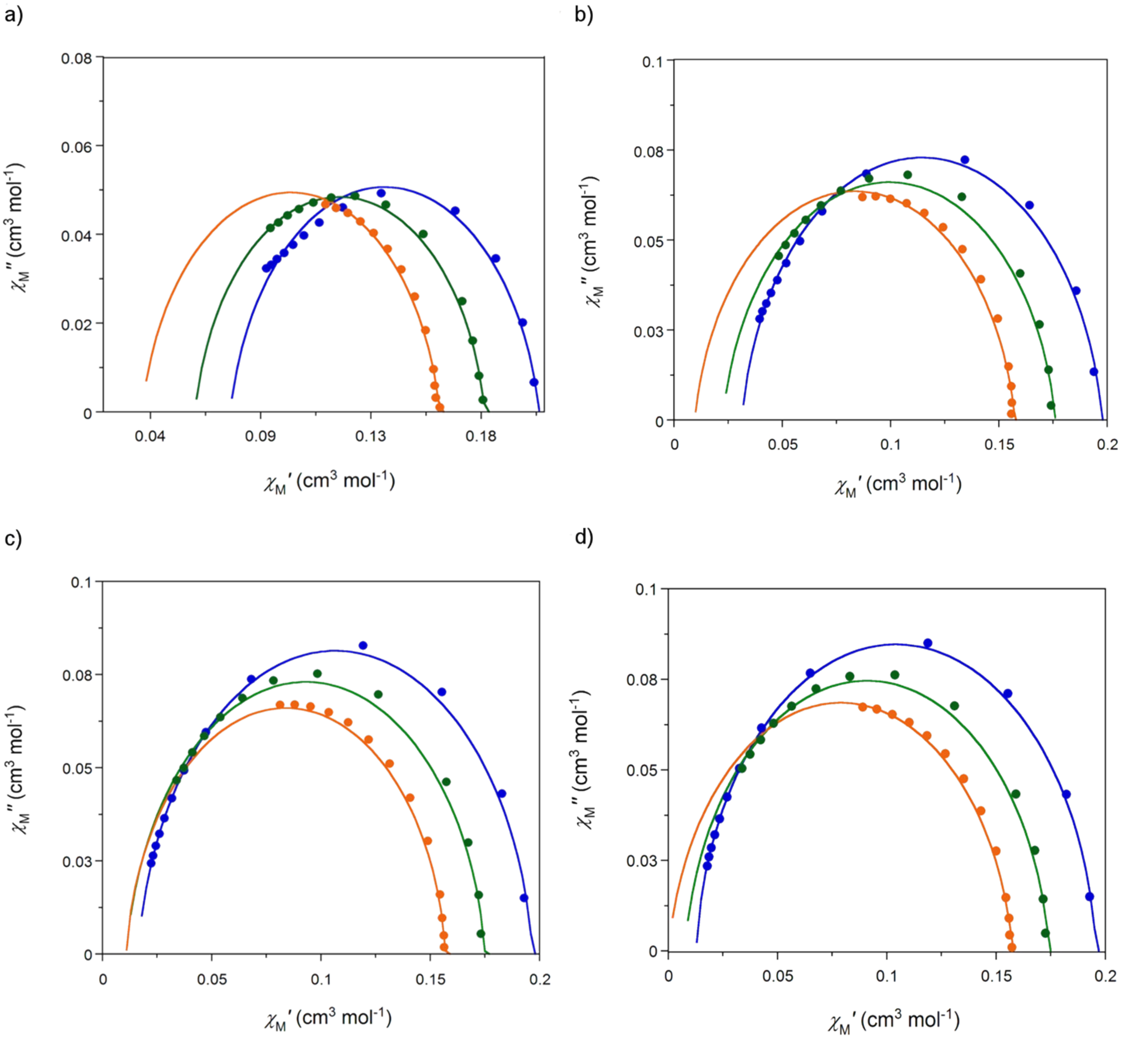

Supplement: Supplementary file 1 [file SC-008-C6SC05188J-s001.pdf]
